# The Gasdermin D N-terminal fragment acts as a negative feedback system to inhibit inflammasome-mediated activation of Caspase-1/11

**DOI:** 10.1073/pnas.2210809119

**Published:** 2022-11-02

**Authors:** Yingchao Hu, Yuying Jiang, Sheng Li, Xiaoqing Ma, Min Chen, Rui Yang, Shuang Wen, Paul N. Moynagh, Bingwei Wang, Gang Hu, Shuo Yang

**Affiliations:** ^a^Department of Immunology, Key Laboratory of Immunological Environment and Disease, Gusu School, State Key Laboratory of Reproductive Medicine, Jiangsu Key Lab of Cancer Biomarkers, Prevention and Treatment, Collaborative Innovation Center for Personalized Cancer Medicine, Center for Global Health, Nanjing Medical University, 211166 Nanjing, China;; ^b^Shanghai Key Laboratory of Maternal Fetal Medicine, Clinical and Translational Research Center of Shanghai First Maternity and Infant Hospital, School of Medicine, Tongji University, 200092 Shanghai, China;; ^c^Department of Pharmacology, Nanjing University of Chinese Medicine, 210023 Nanjing, China;; ^d^Kathleen Lonsdale Institute for Human Health Research, Department of Biology, National University of Ireland Maynooth, Maynooth W23 X021, Ireland;; ^e^Wellcome-Wolfson Institute for Experimental Medicine, Queen's University Belfast, Belfast BT7 1NN, United Kingdom

**Keywords:** Gasdermin D, inflammasome, antiinflammation

## Abstract

Inflammatory pathways usually utilize negative feedback regulatory systems to prevent tissue damage arising from excessive inflammatory response. Whether such negative feedback mechanisms exist in inflammasome activation remains unknown. Gasdermin D (GSDMD) is the pyroptosis executioner of downstream inflammasome signaling. Here, we found that GSDMD, after its cleavage by caspase-1/11, utilizes its RFWK motif in the N-terminal β1-β2 loop to inhibit the activation of caspase-1/11 and downstream inflammation in a negative feedback manner. Furthermore, an RFWK motif-based peptide inhibitor can inhibit caspase-1/11 activation and its downstream substrates GSDMD and interleukin-1β cleavage, as well as lipopolysaccharide-induced sepsis in mice. Collectively, these findings provide a demonstration of the N-terminal fragment of GSDMD as a negative feedback regulator controlling inflammasome activation and a detailed delineation of the underlying inhibitory mechanism.

## Significance

The negative feedback system that tempers inflammasome activation and downstream inflammation remains unknown. To date, there has been intensive study of the role of Gasdermin D as a central player in executing pyroptosis, the cell death pathway downstream of inflammasome activation. We now unexpectedly found that the N-terminal fragment of Gasdermin D can also directly target and inhibit caspase-1/11, the effector caspases in inflammasome pathways, and so suppress downstream triggering of inflammation. We further identify the RFWK motif of the β1-β2 loop of the N-terminal fragment as a critical molecular feature for manifesting the inhibitory effects on caspase-1/11 activity. We also designed a molecule based on the RFWK motif that shows strong antiinflammatory efficacy.

Inflammation is a double-edged sword. In response to infection or tissue injury, it plays a beneficial role in the body to ensure elimination of invasive pathogens and facilitate the repair of damaged tissues (1, 2). However, inflammation sometimes can lose control to become excessive and persistent, which causes pathological damage of tissues and diverse inflammatory diseases, including sepsis, diabetes, cardiovascular disease, inflammatory bowel disease, and neurodegenerative disease (3–8). Thus, the inflammatory response needs to be tightly controlled and resolved in a timely manner. The immune system of our body has evolved to have a variety of negative feedback regulatory mechanisms to precisely control the inflammatory response: for example, IkBα suppressing the NF-κB inflammatory pathway (9), A20 inhibiting TRAF6 signaling (10), IRAK-M inhibiting the Toll-like receptor (TLR) pathway (11), SOCS3 suppressing JAK2/STAT3 signaling (12), and MSK inhibiting the MAPK pathway (13). Overall, these studies highlight the vital role of negative feedback regulation in controlling inflammation.

Inflammasomes are signaling platforms triggered by pathogen-associated molecular patterns and host-derived danger associated molecular patterns, leading to the initiation of inflammatory responses in myeloid cells (14, 15). Upon the recognition of stimuli signals, the core proteins of inflammasome—such as NLRs, AIM2, and Pyrin—are assembled into cytosolic multiprotein complexes together with apoptosis-associated speck-like protein and proinflammatory caspases (caspase-1 and -11), which leads to caspase autoactivation and then processing of interleukin-1β (IL-1β) and IL-18 precursors into their mature forms (16). Moreover, inflammatory caspases cleave Gasdermin D (GSDMD) in its central linker domain to induce the proinflammatory form of programmed cell death, termed pyroptosis (17). Dysregulation of inflammasome activation has been involved in human inflammatory diseases, such as type 2 diabetes, atherosclerosis, gout, multiple sclerosis (MS), and sepsis (18–22). Additionally, gain-of-function mutations in the NLRP3 gene contribute to autoinflammatory diseases, including familial cold autoinflammatory syndrome (FCAS) and Muckle–Wells syndrome (MWS) (23). Although several regulatory mechanisms, such as s-nitrosylation of NLRP3 by nitric oxide (NO) (24), phosphorylation of NLRP3 by PKA (25), and the removal of inflammasomes by autophagy (26), have been identified to suppress inflammasome overactivation, the self-regulating negative feedback mechanisms in inflammasome activation remain to be fully delineated.

GSDMD is a pore-forming protein that drives pyroptosis, downstream of inflammasome activation, and is a member of the human Gasdermin family that also includes GSDMA, GSDMB, GSDMC, GSDME (DFNA5), and GSDMF (DFNB59) (27). Upon inflammasome activation, an L2/L2′ bundle from autoprocessed caspase P20/10 dimers builds an exosite to recognize a hydrophobic pocket in the GSDMD/C-terminal domain (GSDMD-C), that engages the interdomain linker of GSDMD into the catalytic pocket of caspases for cleavage (28, 29). Next, the N-terminal domain of GSDMD (GSDMD-N) is cleaved and released from the autoinhibitory effects of the GSDMD-C domain followed by its conformational change and oligomerization via three protein–protein interfaces of neighboring subunits: α2 helix and β11 strand from one subunit with β2 strand and α3 helix from its neighbor; α1 helix and β3 strand from one subunit with the neighboring α1 helix; and antiparallel β3 and β8 strands from the neighboring subunits (30). Additionally, the GSDMD-N fragments binds lipids through the α1 and α3 helices, the β1-β2 loop, or the β7-β8 sheet and translocate to the plasma membrane to form oligomerized pores, which induces cell lytic death and release of inflammatory mediators, such as IL-1 and IL-18 (31, 32).

In this study, we reveal that the N-terminal fragment of GSDMD can also act as a negative feedback regulator of proinflammatory caspase activation. The RFWK motif of the β1-β2 loop in the N-terminal fragment binds the caspase-1/11 catalytic pocket to inhibit caspase activity, and this serves as a novel regulatory mechanism to negatively control GSDMD-N during inflammasome activation. Furthermore, the administration of a RFWK motif-based peptide inhibitor can inhibit the caspase-1/11 activation and septic death in mice, which may provide important clues for new drug development to target inflammasome caspases.

## Results

### GSDMD Deficiency Exacerbates Inflammasome Activation.

**Fig. 1.**
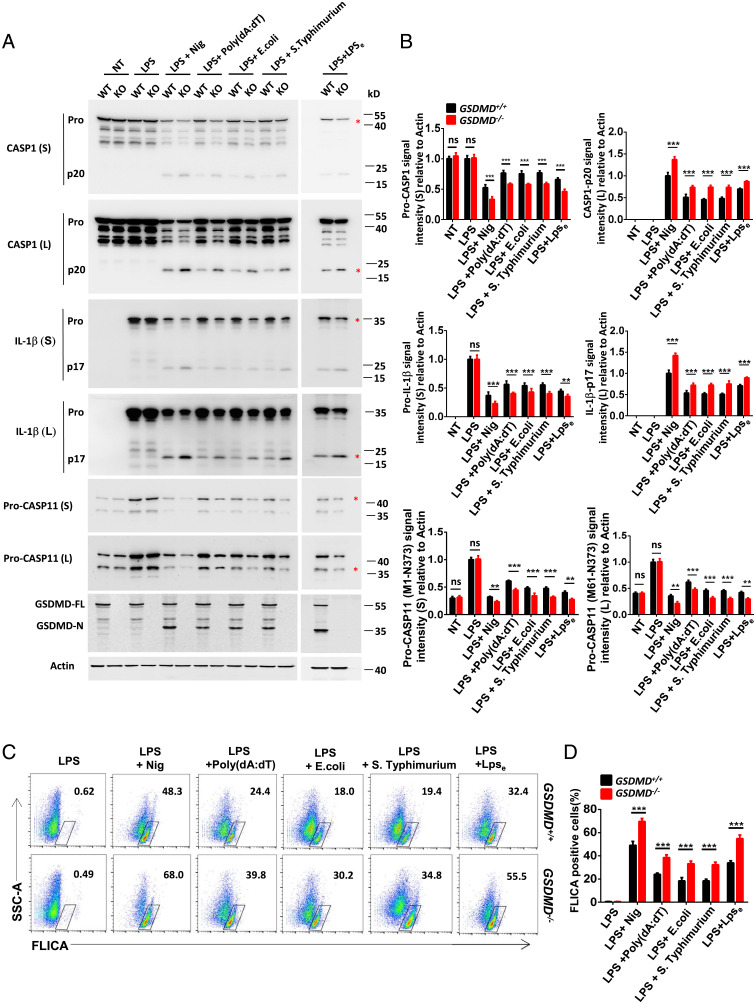
GSDMD deficiency exacerbates inflammasome activation. (*A*) Immunoblot analysis of pooled cell extracts and supernatants from WT and *GSDMD^−/−^* BMDMs primed with LPS (200 ng/mL) for 4 h followed by Nig (10 μM) stimulation for 1 h, or poly(dA:dT) (2 mg/mL) transfection for 16 h, or *S. Typhimurium* (multiplicity of infection [MOI] 30) and *E. coli* (MOI 50) infection for 16 h, or LPS electroporation (2 μg) for 6 h. L, longer exposure; S, Shorter exposure. (*B*) Relative quantification analysis based on grayscale values of *A*. Referring to Actin, the signal intensity of pro-CASP1, pro-IL-1β, and pro-CASP11(M1-N373) were calculated by the gray value of shorter exposure; the CASP1, IL-1β, and pro-CASP11(M61-N373) were calculated by the gray value of longer exposure. An asterisk (*) indicates the bands used for grayscale analysis. (*C* and *D*) Flow-cytometric analysis of CASP1/11 activation probed by FLICA^+^ staining in WT and *GSDMD^−/−^* BMDMs during canonical or noncanonical inflammasome activation as indicated. Data are representative of three independent experiments (*A* and *C*) or are pooled from three independent experiments (*B* and *D*). Error bars show means ± SEM **P < 0.01, ***P < 0.001, ns, not significant. Two-way ANOVA with Sidak’s multiple comparisons test for *B* and *D*.

We initially studied canonical NLRP3 inflammasome activation in *GSDMD^−/−^* bone marrow-derived macrophages (BMDMs) treated with lipopolysaccharide (LPS) plus nigericin (Nig). As expected, GSDMD deficiency blocked LPS-Nig–induced release of lactate dehydrogenase (LDH), Caspase-1(CASP1), and IL-1β, but not tumor necrosis factor (TNF)-α into the supernatants (*SI Appendix*, Fig. S1 *A–D*). However, we unexpectedly observed that the proteolytic processing of CASP1/11 and IL-1β was enhanced in the pooled culture supernatants and cell extracts from *GSDMD^−/−^* BMDMs compared with WT counterparts, as indicated by less pro-CASP1/11 and pro–IL-1β but greater levels of precursor CASP1 (p20) and IL-1β (p17) in LPS-Nig–stimulated *GSDMD^−/−^* cells (Fig. 1 *A* and *B*). This suggested to us that GSDMD may be capable of exercising a negative regulatory effect on NLRP3 inflammasome activation. Next, we examined the role of GSDMD in regulating inflammasome activation by other stimuli, including poly(dA:dT) to activate AIM2-dependent inflammasome, infection with *Salmonella typhimurium* that activates NLRC4 canonical inflammasome, as well as *Escherichia coli* infection and LPS electroporation to engage the noncanonical inflammasome pathway. Like LPS-Nig stimuli, *GSDMD^−/−^* BMDMs exhibited enhanced cleavage of pro-CASP1/11 and pro–IL-1β after poly(dA:dT) treatment, *S. Typhimurium* and *E. coli* infection, and intracellular LPS transfection in pooled samples compared with BMDMs from WT mice (Fig. 1 *A* and *B*), although GSDMD deficiency impaired pyroptosis and the release of CASP1 and IL-1β in supernatants in response to each of these stimuli (*SI Appendix*, Fig. S1 *A–D*). We also utilized the FLICA probe to specially label active caspase-1/11 and found a marked increase of CASP1/11^+^ staining in *GSDMD^−/−^* BMDMs relative to WT cells in response to canonical or noncanonical inflammasome stimuli by fluorescence-activated cell sorting (FACS) analysis (Fig. 1 *C* and *D*).

To further validate the negative regulatory feedback role of GSDMD in human cells, *GSDMD*^−/−^ THP-1 cells were generated by CRISPR-Cas9–mediated targeting. In response to LPS-Nig stimuli, the cleavage of caspase-1 and IL-1β was enhanced greatly in *GSDMD*^−/−^ THP-1 cells, although LDH release was blocked (*SI Appendix*, Fig. S1 *E–G*). Moreover, to clarify that the enhancement effects of GSDMD deficiency on caspase activity and IL-1β processing is direct and not due to an indirect effect of cell lysis, we used the osmoprotectant glycine to prevent pyroptosis-related membrane rupture and so create a “hyperactivation” situation (*SI Appendix*, Fig. S2 *A–C*). We found that under “hyperactivation” conditions, the proteolytic processing of CASP1/11 and IL-1β was still enhanced in the pooled culture supernatants and cell extracts from *GSDMD^−/−^* BMDMs compared with WT counterparts (*SI Appendix*, Fig. S2 *D* and *E*). FACS analysis in glycine-treated unruptured cells also revealed a significant increase of CASP1/11^+^ staining in *GSDMD^−/−^* BMDMs relative to WT cells in response to inflammasome stimuli (*SI Appendix*, Fig. S2 *F* and *G*). Collectively, these data demonstrate that GSDMD, a pyroptosis executioner in the downstream effector phase of inflammasome activation, can feedback to negatively regulate upstream inflammasome activation.

### The N-Terminal Fragment of GSDMD Inhibits Caspase-1/11 Activity during Inflammasome Activation.

To understand how GSDMD regulates CASP1/11 activity, we cotransfected Flag-tagged full-length (mGSDMD-Full), N-terminal (mGSDMD-N), and C-terminal (mGSDMD-C) mouse GSDMD with Myc-tagged mouse CASP1/11(mCASP1/11) and HA-tagged full-length mouse GSDMD (HA-mGSDMD) into HEK293T cells, and then investigated which fragment of GSDMD could affect caspase-1/11–mediated cleavage of HA-mGSDMD. Immunoblot analysis showed that the N-terminal but not the full-length or C-terminal fragment of GSDMD strongly inhibited the cleavage of HA-mGSDMD by CASP1/11 (*SI Appendix*, Figs. S3*A* and S4*A*) with the inhibitory effects of the N-terminal fragment being apparent in a dose-dependent manner (*SI Appendix*, Figs. S3*B* and S4*B*). Similarly, the cleavage of mPro-IL-1β by CASP1 was strongly inhibited by N-terminal fragment of GSDMD (*SI Appendix*, Fig. S4 *C* and *D*). As control, we also used the cotransfection system to analyze the possible role of IL-1β, another substrate of the downstream of inflammasome activation, in affecting CASP1/11 activity, but full length (mIL-1β-Full), N-terminal (mIL-1β-N), and C-terminal (mIL-1β-C) mouse IL-1β showed no such inhibitory effect on the cleavage of HA-mGSDMD by CASP1/11 (*SI Appendix*, Fig. S3*C*). Thus, these data suggest a unique role of GSDMD N-terminal fragment in negatively regulating CASP1/11 activity during inflammasome activation.

**Fig. 2.**
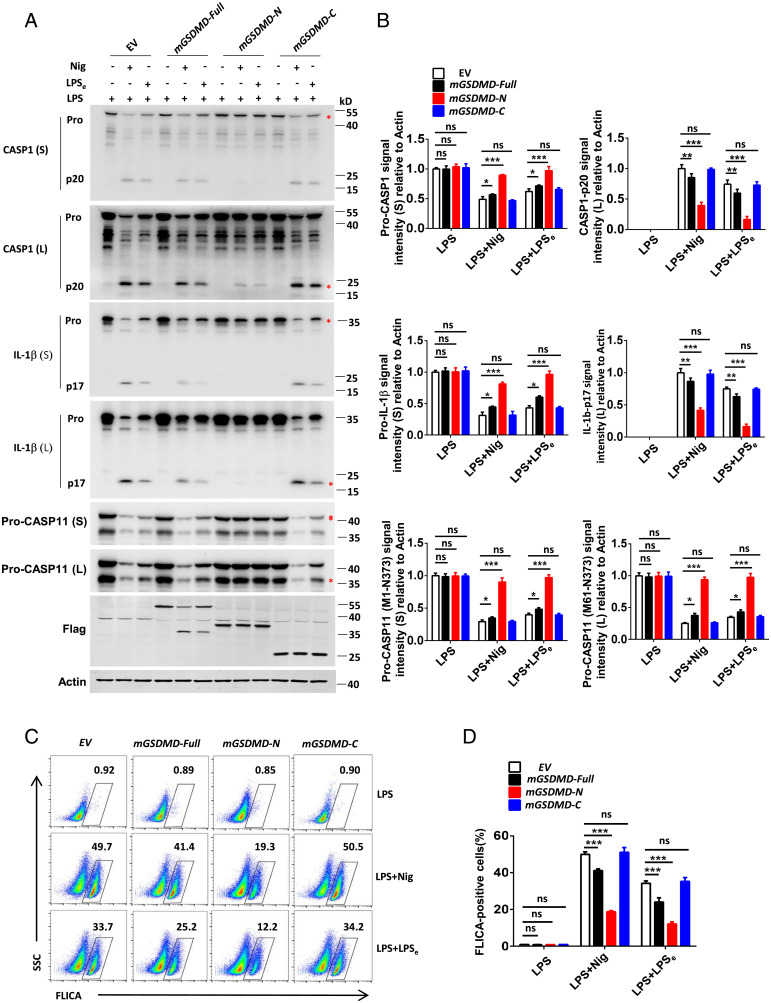
The N-terminal fragment of GSDMD inhibits CASP1/11 activity during inflammasome activation. (*A*) Immunoblot analysis of pooled cell extracts and supernatants from iBMDMs stably expressing full-length (mGSDMD-Full), N-terminal (mGSDMD-N), or C-terminal (mGSDMD-C) fragments of mouse GSDMD during canonical (LPS-Nig, Nig for 1 h) or noncanonical (LPS electroporation for 6 h) inflammasome-triggered processing of pro-CASP1/11 and pro-IL-1β. (*B*) Relative quantification analysis based on grayscale values of *A*. Referring to Actin, the signal intensity of pro-CASP1, pro-IL-1β, and pro-CASP11(M1-N373) were calculated by the gray value of shorter exposure; the CASP1, IL-1β, and pro-CASP11(M61-N373) were calculated by the gray value of longer exposure. An asterisk (*) indicates the bands used for grayscale analysis. (*C* and *D*) Flow-cytometric analysis of CASP1/11 activation probed by FLICA^+^ staining in iBMDMs stably expressing mGSDMD-Full, mGSDMD-N, and mGSDMD-C upon inflammasome activation as indicated. Data are representative of three independent experiments (*A* and *C*) or are pooled from three independent experiments (*B* and *D*). Error bars show means ± SEM *P < 0.05, **P < 0.01, ***P < 0.001; ns, not significant. Two-way ANOVA with Sidak’s multiple comparisons test for *B* and *D*.

To further validate this inhibitory function of the GSDMD N-terminal fragment, immortalized BMDMs (iBMDMs) stably expressing mGSDMD-Full, mGSDMD-N, or mGSDMD-C fragments were generated by use of the pLV lentivirus system. Notably, the expression of exogenous GSDMD-N in stable cell lines is closer to endogenous levels of GSDMD. While this stable expression of exogenous GSDMD-N does not cause cytotoxicity to cells, more toxic effects are observed when cells are stimulated with relevant stimuli (*SI Appendix*, Fig. S5*B*). Immunoblot analysis showed that the stably expressing mGSDMD-N but not mGSDMD-Full or mGSDMD-C fragment in BMDMs significantly blocked both canonical (as stimulated by LPS-Nig) and noncanonical (as stimulated by LPS electroporation)-triggered processing of pro-CASP1/11 and pro-IL-1β (Fig. 2 *A* and *B*), although mGSDMD-N–expressing iBMDMs exhibited greater release of pro-CASP1, pro-IL-1β, and LDH into supernatant in response to inflammasome stimuli (*SI Appendix*, Fig. S5 *A* and *C*). FACS analysis also revealed that the stably expressing mGSDMD-N fragment markedly reduced the staining of FLICA probe in iBMDMs treated with LPS-Nig and LPS electroporation (Fig. 2 *C* and *D*). We also reconstructed the full-length (hGSDMD-Full), N terminus (hGSDMD-N) and C terminus (hGSDMD-C) of human GSDMD into THP-1 cells, and similarly observed that the inhibition of caspase-1/4 autoprocessing and IL-1β cleavage was only evident in THP1 cells stably expressing the hGSDMD-N fragment under the stimulation of LPS-Nig (*SI Appendix*, Fig. S5 *D–I*). These data add further support to our hypothesis that GSDMD inhibits CASP1/11 activity though its N-terminal fragment and so act as feedback mechanism to suppress excessive inflammasome activation.

### The Expression of GSDMD N-Terminal Fragment in Peripheral Myeloid Cells Suppresses Neuroinflammation and Pathogenesis of Experimental Autoimmune Encephalomyelitis.

**Fig. 3.**
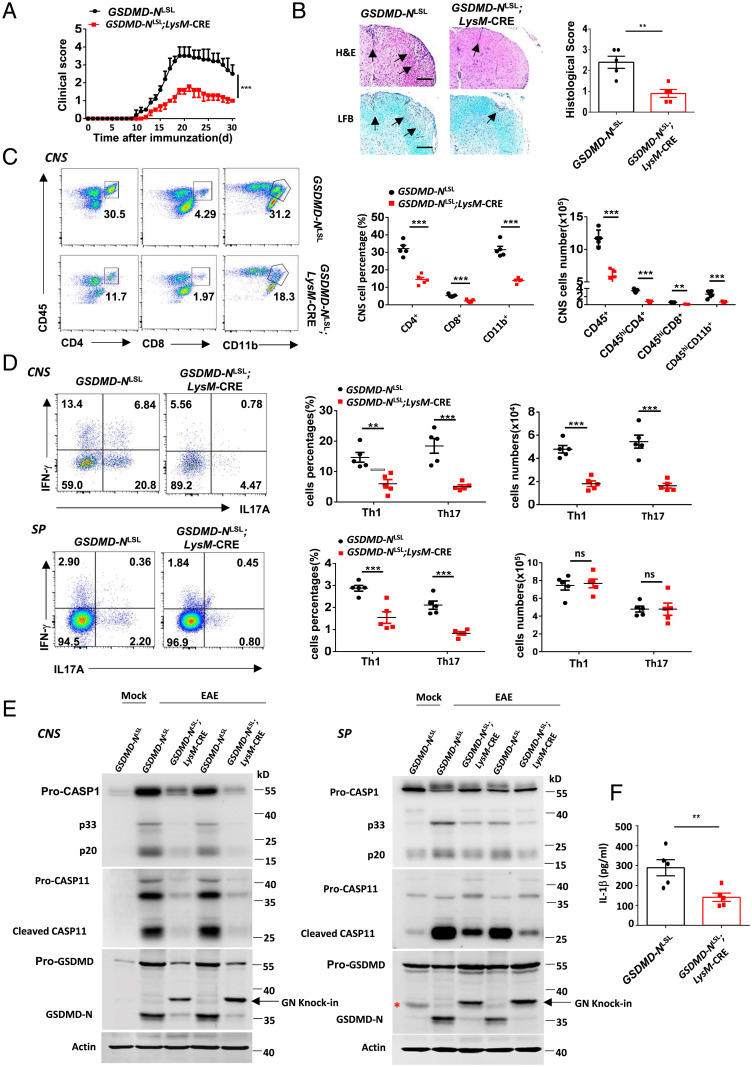
The expression of GSDMD N-terminal fragment in peripheral myeloid cells suppresses neuroinflammation and pathogenesis of EAE. (*A*) Mean clinical score of age-matched female *GSDMD-N*^LSL^*;LysM-*Cre and littermate control mice subjected to MOG_35–55_-induced EAE (*n* = 5 mice per group). (*B*) Representative images and histological scores of H&E staining and LFB staining of spinal cord sections from mice in *A*. Inflammatory cell infiltration and demyelination indicated by arrow. (Scale bars, 200 μm.) Histological scores: 0, no inflammatory cell infiltration and no demyelination; 1, slight inflammatory cell infiltration or demyelination observed; 2, moderate inflammatory cell infiltration or demyelination in several spots; 3, substantial inflammatory cell infiltration and large area of demyelination. (*C*) Flow-cytometric analysis of immune cells (including CD45^+^CD4^+^ T cells, CD45^+^CD8^+^ T cells, and CD45^+^CD11b^+^ monocytes) infiltrated to the spinal cord and brain (CNS) of MOG_35–55_-immunized *GSDMD-N*^LSL^*;LysM-*Cre and littermate control mice at day 18 after immunization. Data are presented as a representative plot and summary graph of quantified percentage and absolute cell numbers. (*D*) Flow-cytometric analysis of Th1 (IFN-γ^+^) and Th17 (IL-17A^+^) cells from CD4^+^ T cells in CNS (*Upper*) and spleen (*Lower*) of MOG_35–55_-immunized *GSDMD-N*^LSL^*;LysM-*Cre and littermate control mice at day 18 after immunization. Data are presented as a representative plot, quantified percentage, and absolute cell numbers. (*E*) Immunoblot analysis of the cleavage of CASP1/11 and endogenous GSDMD in CNS (*Left*) and spleen (*Right*) of MOG_35–55_-immunized *GSDMD-N*^LSL^*;LysM-*Cre mice compared with control mice at day 18 after immunization. An asterisk (*) indicates nonspecific bands. (*F*) ELISA analysis of IL-1β in sera collected in MOG_35–55_-immunized *GSDMD-N*^LSL^;*LysM-*Cre and littermate control mice at day 18 after immunization. Data are pooled from three independent experiments (*A–F*). Error bars show means ± SEM *P < 0.05, **P < 0.01, ***P < 0.001; ns, not significant. Unpaired *t* test for *A, B*, and *F*, and multiple unpaired *t* test for *C* and *D*.

We next investigated whether the N-terminal fragment of GSDMD could inhibit NLRP3 inflammasome in vivo. To this end, we generated a knockin mouse by inserting 3×Flag-GSDMD-N fragment with loxp-flanked stop cassette into the Rosa26 locus, and then crossed them with *LysM*-Cre mice to delete the stop cassette and produce myeloid-specific GSDMD-N fragment expressing mice (*GSDMD-N*^LSL^*;LysM-*Cre) (*SI Appendix*, Fig. S6 *A* and *B*). The expression of exogenous GSDMD-N in mice is comparable with endogenous levels of GSDMD (Fig. 3*E*) and the development and immunophenotype of the mice were normal. However, consistent with iBMDMs expressing mGSDMD-N, primary BMDMs from *GSDMD-N*^LSL^*;LysM-*Cre mice exhibited significantly decreased cleavage of CASP1 and IL-1β but more pyroptosis in response to LPS-Nig and LPS electroporation stimuli compared with control BMDMs from littermates (*SI Appendix*, Fig. S6 *C* and *D*). We performed LPS-induced sepsis model on *GSDMD-N*^LSL^*;LysM-*Cre mice and found that the knockin mice were more susceptible to sepsis (*SI Appendix*, Fig. S6*E*). This is in line with the findings of more pyroptosis in primary knockin BMDMs in response to stimuli and also consistent with previous reports that GSDMD-mediated pyroptosis is primarily responsible for LPS-induced sepsis (33, 34).

Given the sensitive response of knockin mice in LPS-sepsis, and inflammasome activation heavily required for the development of experimental autoimmune encephalomyelitis (EAE) (35, 36), we thus performed the EAE model on *GSDMD-N*^LSL^*;LysM-*Cre mice to further evaluate the inhibitory effect of GSDMD-N fragment on inflammasome activation in vivo. Notably, *GSDMD-N*^LSL^*;LysM-*Cre mice were more resistant to EAE development relative to their littermate controls, as indicated by less severe clinical and histopathological scores (Fig. 3 *A* and *B*). Furthermore, FACS revealed a marked reduction of T cells (CD45^+^CD4^+^ and CD45^+^CD8^+^), and myeloid cells and activated microglia cells (CD45^high^CD11b^+^) in the central nervous system (CNS) of *GSDMD-N*^LSL^*;LysM-*Cre mice during EAE (Fig. 3*C*). Additionally, the percentages and absolute numbers of both Th1 and Th17 cells were greatly reduced in the CNS of *GSDMD-N*^LSL^*;LysM-*Cre mice compared with controls. The percentages of Th1 and Th17 cells were also markedly reduced in the spleen of *GSDMD-N*^LSL^*;LysM-*Cre mice, while the absolute numbers were comparable with control mice (Fig. 3*D*). Immunoblot analysis demonstrated the greatly decreased processing of CASP1/11 and GSDMD in both CNS and peripheral spleen of *GSDMD-N*^LSL^*;LysM-*Cre mice compared with control mice during the peak of EAE (Fig. 3*E*). The levels of IL-1β in sera were also lower in *GSDMD-N*^LSL^*;LysM-*Cre mice (Fig. 3*F*). All these data further suggest that the GSDMD-N fragment in peripheral myeloid cells blocks inflammasome activation and suppresses neuroinflammation associated with EAE.

### The N-Terminal Fragment of GSDMD Binds Caspase-1/11 Catalytic Domain via Its RFWK Motif in the β1-β2 Loop.

To understand the structural basis to the inhibitory effects of the GSDMD-N fragment on CASP1/11 activity, we next explored the possible binding between GSDMD-N fragment and CASP1/11 catalytic domain (p20/p10 for CASP1 and p22/p10 for CASP11) by crystal structure docking analysis. The murine CASP1 (p20/p10) model was generated based on the structure of human CASP1 (C285A)-p20/p10 (PDB ID code 6KN0) (28) by homology-modeling (SWISS-MODEL). The caspase-11 (C254A)-p22/p10 structure (PDB ID code 6KMU) (28) was used as murine CASP11 (p22/p10) model. The inactivating conformation of murine GSDMD-N fragment in full-length mGSDMD with cleavage linker (mGSDMD-N_i_) was generated based on the structure of full-length murine GSDMD (PDB ID code 6N9N) (31) by the program Modeler (*SI Appendix*, Fig. S7*A*). Moreover, the activating conformation of murine GSDMD-N fragment with flexible cleavage linker (mGSDMD-N_a_) was constructed based on the activating structure of the murine GSDMA3 (PDB ID code 6CB8) (30) and human GSDMD (PDB ID code 6VFE) (32). Next, we performed the random docking analysis by using ZDOCK SERVER. The results demonstrated that the preferred interaction between GSDMD-N fragment and CASP1/11 catalytic subunits is via the GSDMD-N-terminal β1-β2 loop binding to the CASP1/11 catalytic pocket, as indicated by Rank 1–2, 4, and 5 in the top 10 docking interactions of mGSDMD-N_i_ and CASP1 (p20/p10), Rank 1–2 and 5 in the top 10 docking interactions of mGSDMD-N_i_ and CASP11 (p22/p10), Rank 1–4 and 6 in the top 10 docking interactions of mGSDMD-N_a_ and CASP1 (p20/p10), as well as Rank 1–3 in the top 10 docking interactions of mGSDMD-N_a_ and CASP11 (p22/p10) (*SI Appendix*, Fig. S7 *B–E*). Similar docking results were obtained by the Schrödinger analysis. Next, we chose Rank 1 docking structures of mGSDMD-N_i_ and CASP1/11 catalytic domain to further analyze their detailed binding interface.

**Fig. 4.**
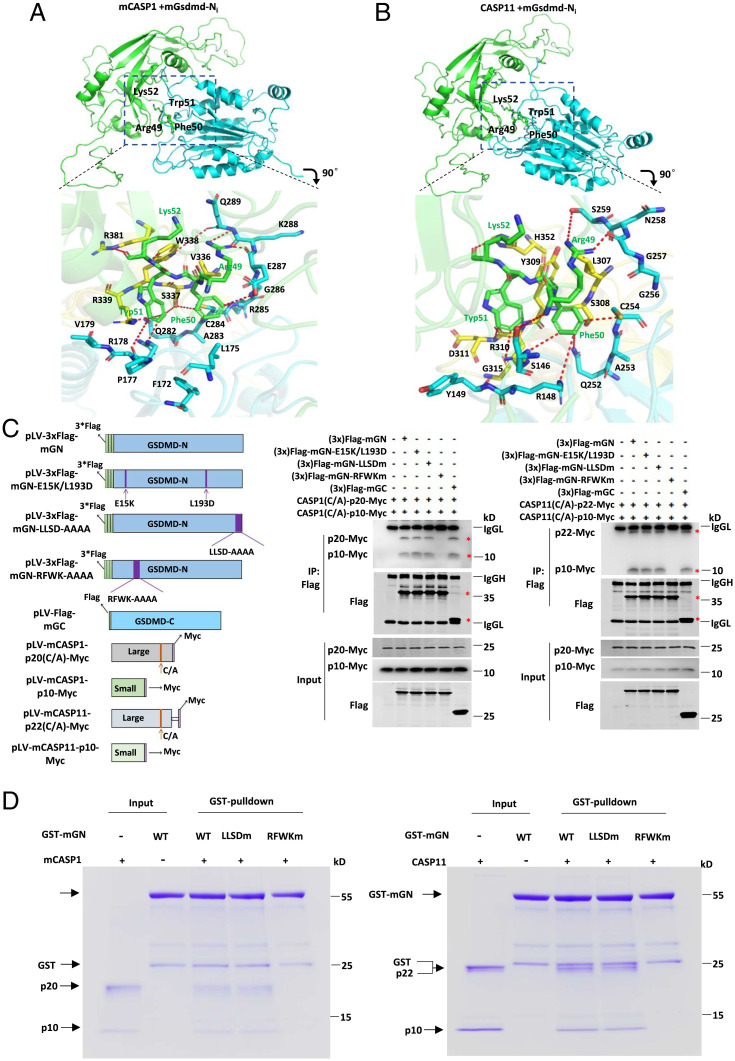
The RFWK motif in the β1-β2 loop of the N-terminal fragment of GSDMD is critical for binding to the catalytic domain of caspase-1/11. (*A*) Rank 1 docking structure of mGSDMD-N_i_ and mCASP1(p20/p10) and detailed binding interface. (*Upper*) mGSDMD-N_i_ and mCASP1(p20/p10) are colored green and cyan, respectively. (*Lower*) RFWK residues of mGSDMD-N_i_ are colored green, p20 and p10 subunits of mCASP1 are colored cyan and yellow, respectively. (*B*) Rank 1 docking structure of mGSDMD-N_i_ and mCASP11(p22/p10) and detailed binding interface. (*Upper*) mGSDMD-N_i_ and mCASP1(p20/p10) are colored green and cyan, respectively. (*Lower*) RFWK residues of mGSDMD-N_i_ are colored green, p22 and p10 subunits of mCASP11 are colored cyan and yellow, respectively. (*C*, *Left*) domain architecture of Flag-tagged mGSDMD-N fragment and mGSDMD-N fragment mutants (mGSDMD-N_E15K/L193Dm_, mGSDMD-N_RFWKm_, and mGSDMD-N_LLSDm_) and mGSDMD-C fragment and Myc-tagged CASP1/11(C/A) p20/p10 or p22/p10 subunits. (*Right* and *Center*) Immunoblot analysis of Myc and Flag proteins in immunoprecipitated Flag and lysate (input) samples from HEK293T cells transfected with constructs encoding Myc-tagged mouse CASP1/11(C/A)-p20/p10 and Flag-tagged mouse GSDMD-N fragment and mGSDMD-N mutants (mGSDMD-N_RFWKm_ and mGSDMD-N_LLSDm_). (*D*) In vitro glutathione S-transferase (GST) pull-down assay. mGSDMD-N, mGSDMD-N_RFWKm_, or mGSDMD-N_LLSDm_ was used to pull down the enzyme inactive p20/p10 or p22/p10 form of CASP1/11.

Close inspection of the binding interface between mGSDMD-N_i_ and CASP1 (p20/p10) revealed the _49_RFWK_52_ residues (Arg49, Phe50, Trp51, Lys52) in the mGSDMD-N_i_ β1-β2 loop were modeled to dock onto the catalytic groove of mCASP1 formed by L1 to L4 loops, with such docking being mediated by hydrophobic interactions and hydrogen bonds. In brief, Phe50 in the β1-β2 loop of mGSDMD-N_i_ forms hydrophobic contacts with Leu175 of L1, Ala283 and Gly286 of L2, and Val336 and Trp338 of L3 from mCASP1. Trp51 in the β1-β2 loop forms hydrophobic interactions with Phe172 and Val179 of L1, Ala283 of L2, and Val336 and Trp338 of L3. The hydrophobic interactions are further strengthened by nearby hydrogen bonds between RFWK and the residues of L1-4. Arg49 from mGSDMD-N_i_ forms hydrogen bonds with Glu287, Lys288, and Gln289 of L2, and Trp338 of L3 in mCASP1. Phe50 of mGSDMD-N_i_ forms hydrogen bonds with Cys284, Arg285, and Glu287 of L2, and Ser337 of L3. Trp51 of mGSDMD-N_i_ forms hydrogen bonds with Pro177 and Arg178 of L1, Gln282 of L2, and Ser337 and Arg339 of L3. Lys52 from mGSDMD-N_i_ forms hydrogen bonds with Trp338 of L3 and Arg381 of L4 (Fig. 4*A*). Similar to mGSDMD-N_i_ binding to CASP1 (p20/p10), the RFWK residues of mGSDMD-N_i_ were also docked onto the catalytic center of mCASP11 to form a binding interface via hydrophobic interactions and hydrogen bonds. Phe50 in the β1-β2 loop forms hydrophobic contacts with Ala253, Gly256, and Gly257 of L2, and Leu307 and Tyr309 of L3. Trp51 forms hydrophobic interactions with Tyr149 of L1, and Leu307, Tyr309, and Gly315 of L3. Hydrophilic interactions also contribute to the extensive interactions. Arg49 residue forms hydrogen bonds with Ser146 of L1, and Asn258 and Ser259 of L2. Phe50 forms hydrogen bonds with Arg148 of L1, Gln252 and Cys254 of L2, and Ser308 and Arg310 of L3. Trp51 forms hydrogen bonds with Arg310 and Asp311 of L3. Lys52 forms hydrogen bonds with His352 of L4 (Fig. 4*B*).

To further affirm that the RFWK motif structure is required for the interaction of the GSDMD-N fragment with CASP1/11 catalytic subunits, we performed alanine substitution on the RFWK motif to generate a Flag-tagged mGSDMD-N_RFWKm_ mutant, and then conducted CASP1/11 catalytic subunits interaction studies by coimmunoprecipitation analysis in HEK293T cells. mGSDMD-C was used as a positive control for interaction with the caspase subunits. Notably, mGSDMD-N, but not the mGSDMD-N_RFWKm_, mutant was shown to coimmunoprecipitate with the p20/p10 and p22/p10 subunits. Additionally, a mutant of mGSDMD-N with mutation of its oligomerization site (mGSDMD-N_E15K/L193Dm_) or a form of mGSDMD with mutation of its cleavage-site tetrapeptide (mGSDMD-N_LLSDm_) still retained binding of the GSDMD-N fragment to the CASP1/11 catalytic subunits (Fig. 4*C*). To further investigate the direct association of the GSDMD-N fragment with CASP1/11 via the RFWK motif, GST-mGSDMD-N, GST-mGSDMD-N_RFWKm_ and GST-mGSDMD-N_LLSDm_ recombinant proteins were used, respectively, to pull-down the enzymatically inactive p20/p10 or p22/p10 forms of CASP1/11. We found that mGSDMD-N and mGSDMD-N_LLSDm_, but not mGSDMD-N_RFWKm_ protein, could directly bind to CASP1/11(C/A) p20/p10 or p22/p10 forms (Fig. 4*D*). Overall, these data suggest that the N-terminal fragment of GSDMD directly binds to CASP1/11 catalytic domain through its RFWK motif.

### The N-Terminal Fragment of GSDMD Inhibits Caspase-1/11 Activity and Inflammasome Activation via Its RFWK Motif.

**Fig. 5.**
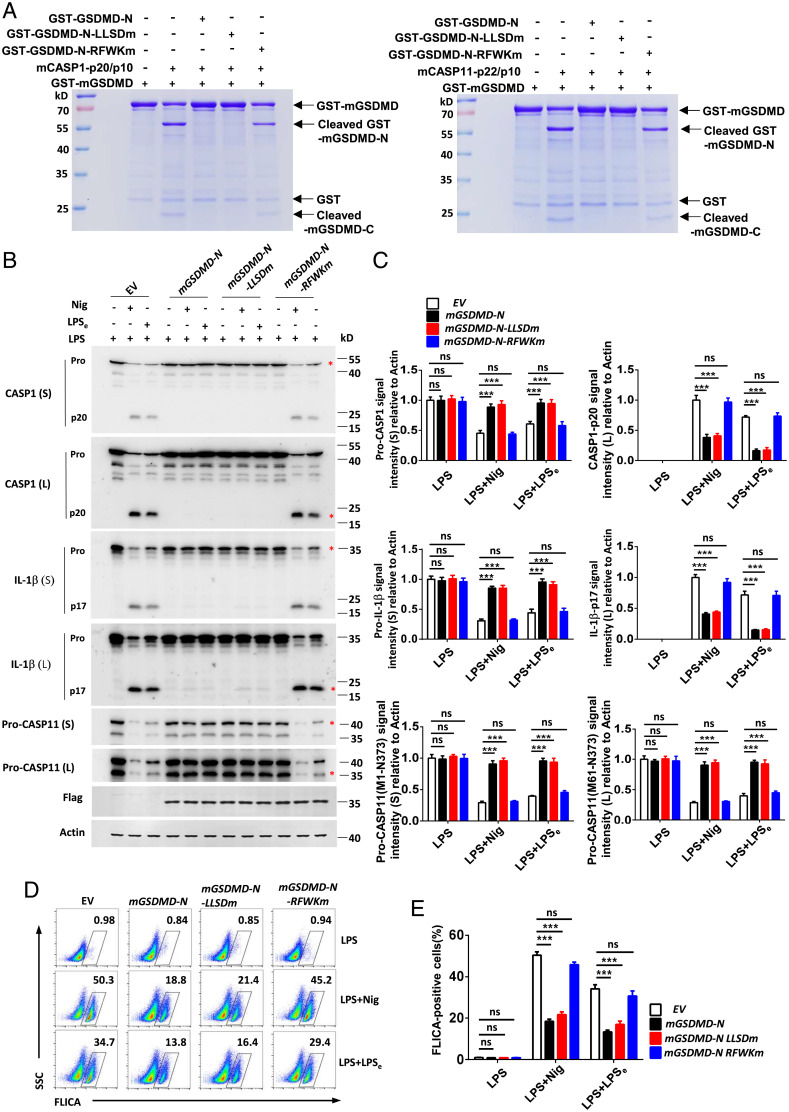
The RFWK motif in the N-terminal fragment of GSDMD is critical for inhibiting caspase-1/11 activity and inflammasome activation. (*A*) Recombinant full-length GSDMD protein cleaved by CASP1/11 in the presence of mGSDMD-N, mGSDMD-N_RFWKm_, and mGSDMD-N_LLSDm_ protein in vitro. (*B*) Immunoblot analysis of pooled cell extracts and supernatants from iBMDMs stably expressing mGSDMD-N, mGSDMD-N_LLSDm_, and mGSDMD-N_RFWKm_ during canonical LPS-Nig (Nig for 1 h) or noncanonical LPS electroporation (6 h)-triggered processing of pro-CASP1/11 and pro-IL-1β. (*C*) Relative quantification analysis based on grayscale values of *B*. Referring to Actin, the signal intensity of pro-CASP1, pro-IL-1β, and pro-CASP11(M1-N373) were calculated by the gray value of shorter exposure; the CASP1, IL-1β, and pro-CASP11(M61-N373) were calculated by the gray value of longer exposure. An asterisk (*) indicates the bands used for grayscale analysis. (*D* and *E*) Flow-cytometric analysis of CASP1/11 activation probed by FLICA^+^ staining in iBMDMs stably expressing mGSDMD-N, mGSDMD-N_LLSDm_, and mGSDMD-N_RFWKm_ upon inflammasome activation as indicated. Data are pooled from three independent experiments (*A–E*) or representative of three independent experiments (*A, B*, and *D*). Error bars show means ± SEM ***P < 0.001; ns, not significant. Two-way ANOVA with Sidak’s multiple comparisons test for *C* and *E*.

Given that our data strongly indicate that the GSDMD-N fragment utilizes its RFWK motif to bind the CASP1/11 catalytic domain, we next examined if the RFWK motif was required to manifest the negative inhibitory effects of GSDMD-N fragment on CASP1/11 activity. Using a cotransfection system, we found that mGSDMD-N, mGSDMD-N_E15K/L193Dm_, and mGSDMD-N_LLSDm_, but not mGSDMD-N_RFWKm_, strongly inhibit the cleavage of HA-mGSDMD by CASP1/11 (*SI Appendix*, Fig. S8). Additionally, recombinant mGSDMD-N and mGSDMD-N_LLSDm_, but not mGSDMD-N_RFWKm_ protein, could inhibit the cleavage of full-length GSDMD by CAPS1/11 enzyme in vitro (Fig. 5*A*). These data demonstrate a critical role for the RFWK motif in mediating the inhibitory role of N-terminal fragment of GSDMD on CASP1/11 activity. Furthermore, immunoblot analysis showed that stably expressing mGSDMD-N and mGSDMD-N_LLSDm_, but not mGSDMD-N_RFWKm_ in iBMDMs, significantly blocked both canonical and noncanonical inflammasome-triggered cleavage of pro-CASP1/11 and pro-IL-1β (Fig. 5 *B* and *C* and *SI Appendix*, Fig. S9*B*). However, mGSDMD-N_RFWKm_–expressing cells exhibited less release of pro-CASP1, pro-IL-1β, and LDH than cells expressing mGSDMD-N or mGSDMD-N_LLSDm_ after stimuli (*SI Appendix*, Fig. S9 *A* and *C*). This is consistent with the lower ability of the GSDMD-N mutant in the β1-β2 loop than that of WT to anchor the lipid to form the pores in plasma membrane and induce pyroptotic cell death (31). FACS analysis also revealed that mGSDMD-N and mGSDMD-N_LLSDm_, but not mGSDMD-N_RFWKm_, inhibited the staining of active CASP1/11 enzyme in iBMDMs treated with LPS-Nig and LPS electroporation (Fig. 5 *D* and *E*).

To further exclude the contribution of any processing of endogenous GSDMD, we next reconstituted GSDMD-deficient iBMDMs with different forms of GSDMD to examine their effect on CASP1/11 activity and inflammasome activation. Therefore, we generated *GSDMD^−/−^* iBMDMs stably expressing mGSDMD-Full, mGSDMD-N, mGSDMD-C, and mGSDMD-N-RFWKm. Immunoblot analysis showed that the stably expressing mGSDMD-N but not mGSDMD-Full, mGSDMD-C or mGSDMD-N-RFWKm in *GSDMD^−/−^* iBMDMs significantly blocked both LPS-Nig and electroporated LPS-triggered processing of pro-CASP1/11 and pro-IL-1β (*SI Appendix*, Fig. S10 *A* and *B*). FACS analysis also revealed that mGSDMD-N markedly reduced the staining of FLICA probe of active CASP1/11 enzyme in *GSDMD^−/−^* iBMDMs treated with LPS-Nig and LPS electroporation (*SI Appendix*, Fig. S10 *C* and *D*). Meanwhile, we demonstrated the release of LDH, CASP1, and IL-1β under stimuli were rescued in *GSDMD^−/−^* iBMDMs stably expressing mGSDMD-Full and mGSDMD-N, whereas mGSDMD-C and mGSDMD-N-RFWKm expressing *GSDMD^−/−^* iBMDMs exhibited no release of LDH, CASP1, and IL-1β (*SI Appendix*, Fig. S10 *E* and *F*). Additionally, we generated *GSDMD^−/−^* iBMDMs stably expressing full-length RFWK mutant (mGSDMD-Full_RFWKm_) and examined canonical or noncanonical inflammasome activation. We found the RFWK mutant blocked the release of LDH, CASP1, and IL-1β into the supernatants (*SI Appendix*, Fig S11 *E* and *F*), which is consistent with previous reports showing the β1-β2 loop is indispensable for lipid binding and pyroptosis (31, 32). However, the processing of CASP1/11 and IL-1β was significantly enhanced in the pooled culture supernatants and cell extracts from *GSDMD^−/−^* iBMDMs reconstituted with the GSDMD RFWK mutant compared with WT counterparts (*SI Appendix*, Fig. S11 *A–D*). Thus, all these data further suggest that the N-terminal fragment of GSDMD inhibits caspase-1/11 activity and inflammasome activation in a manner that is critical, dependent on its RFWK motif.

### A RFWK Motif-Based Peptide Inhibitor Suppresses Inflammasome Activation and LPS-Induced Sepsis in Mice.

**Fig. 6.**
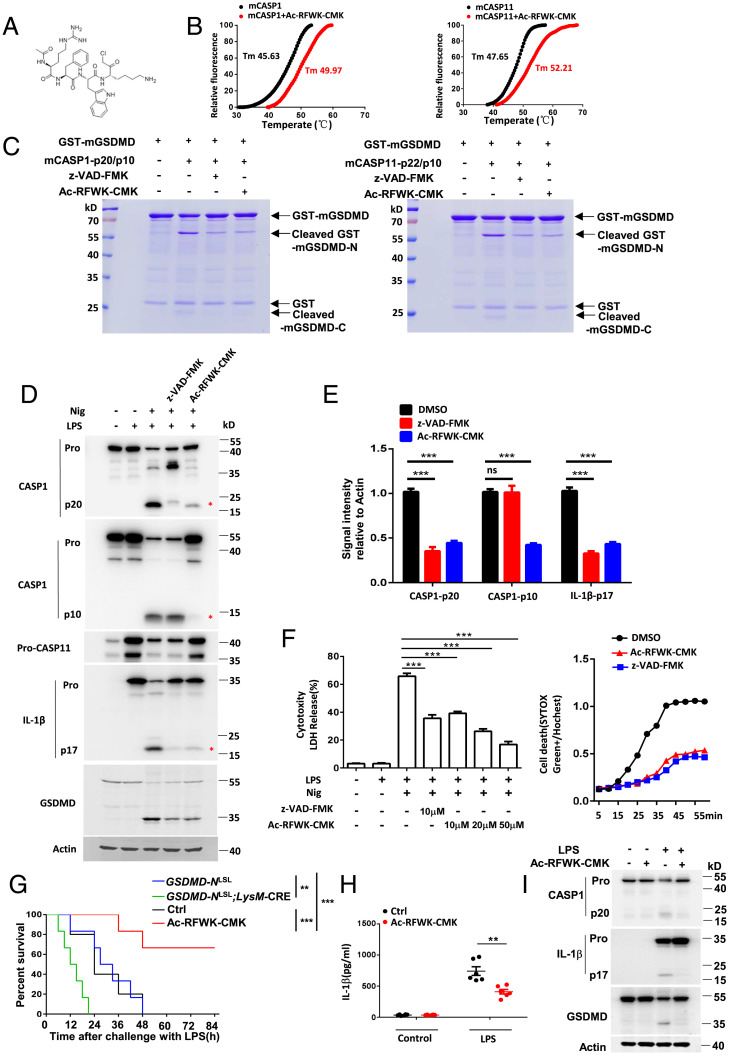
RFWK motif-based inhibitor Ac-RFWK-CMK suppresses inflammasome activation and LPS-induced sepsis in mice. (*A*) Chemical structure of Ac-RFWK-CMK inhibitor. (*B*) Thermal shift assay between the Ac-RFWK-CMK inhibitor and the mouse CASP1/11(C/A) catalytic domain. (*C*) Recombinant full-length GSDMD protein cleaved by CASP1/11 in the presence of Ac-RFWK-CMK (10 μΜ) and z-VAD-FMK (10 μΜ) in vitro. (*D*) Immunoblot analysis of pooled cell extracts and supernatants from primary wild-type BMDMs in the presence of Ac-RFWK-CMK (10 μΜ) upon NLRP3 inflammasome activation (LPS-Nig, Nig for 1 h). (*E*) Relative quantification analysis based on grayscale values of *D*. An asterisk (*) indicates the bands used for grayscale analysis. (*F*) Effects of Ac-RFWK-CMK inhibitor on NLRP3 inflammasome-mediated pyroptosis as measured by LDH release and permeability of cells to SYTOX green in BMDMs. (*G*) Integration of the survival data from *GSDMD-N*^LSL^;*LysM-*Cre mice and mice treated with Ac-RFWK-CMK inhibitor (25 mg/kg) before challenge with LPS (25 mg/kg) (*n* = 6). (*H*) ELISA analysis of IL-1β in sera of mice treated with vehicle or Ac-RFWK-CMK inhibitor (*n* = 6 mice per group) 8 h after 25 mg/kg LPS challenge. (*I*) Immunoblot analysis of the cleavage of CASP1, IL-1β, and GSDMD in peritoneal macrophages from septic mice after 6 h of LPS challenge. Data are pooled from three independent experiments (*B* and *E–H*) or are representative of three independent experiments (*C, D*, and *I*). Error bars show means ± SEM **P < 0.01, ***P < 0.001, ns, not significant. Two-way ANOVA with Sidak’s multiple comparisons test for *E*; one-way ANOVA with Dunnett’s multiple comparisons test for LDH assay and unpaired *t* test for SYTOX Green assay (*F*); log rank (Mantel-Cox) test for *G* and unpaired *t* test for *H*.

Given the critical importance of the RFWK motif in manifesting the inhibitory effects of the N-terminal fragment of mGSDMD-N on CASP1/11 activity, we designed an Ac-RFWK-CMK peptide that contains an acetyl moiety and chloromethyl ketone, like other caspase inhibitors, to target inflammatory caspases (Fig. 6*A*). The thermal shift assay showed a marked shift of the melting temperature for the recombinant CASP1/11(C/A) catalytic domain in the presence of the inhibitor, suggesting the inhibitor can bind to CASP1/11 enzyme (Fig. 6*B*). Moreover, the cleavage of recombinant mGSDMD by CASP1/11 enzyme was strongly inhibited in the presence of Ac-RFWK-CMK to similar extent as the pan-caspase inhibitor z-VAD-FMK (Fig. 6*C*). Immunoblot analysis showed that Ac-RFWK-CMK could inhibit the cleavage of pro-CASP1 to p20/p10, pro-IL-1β to p17, and GSDMD to p30 in BMDMs after LPS-Nig stimulation. Notably, while pan-caspase inhibitor z-VAD-FMK has little effect on LPS-Nig–induced CASP1-p10 cleavage, it blocked formation of the 20-kDa fragment and significantly inhibited the cleavage of IL-1β and GSDMD (Fig. 6 *D* and *E*). Additionally, like the pan-caspase inhibitor z-VAD-FMK, Ac-RFWK-CMK impaired LPS-Nig–induced cell death in macrophages (Fig. 6*F*). To investigate the in vivo inhibitory effect of Ac-RFWK-CMK, we examined LPS-induced sepsis in C57BL/6 mice. Mice were administered intraperitoneally with vehicle or the Ac-RFWK-CMK inhibitor (25 mg/kg) before challenge with LPS (25 mg/kg) and then evaluated for survival. We observed that LPS challenge resulted in the death of all mice within 48 h, but Ac-RFWK-CMK treatment reduced the lethal rate to about 30% (Fig. 6*G*). The levels of IL-1β in the sera of mice treated with Ac-RFWK-CMK inhibitor were strongly decreased at 12 h after LPS challenge compared to vehicle-treated control mice (Fig. 6*H*). Moreover, Ac-RFWK-CMK strongly inhibited the cleavage of CASP1, GSDMD, and IL-1β in peritoneal macrophages collected at 6 h post-LPS challenge (Fig. 6*I*).

To discount a role for the CMK moiety in mediating the inhibitory effects, we also evaluated the effect of the unmodified parent RFWK peptide (Ac-RFWK) on caspase activity and found that Ac-RFWK could significantly inhibit pyroptosis and inflammasome activation in BMDMs after LPS-Nig stimulation, with the inhibitory effects being comparable to the effect of Ac-RFWK-CMK (*SI Appendix*, Fig. S12 *A–C*). Additionally, Ac-RFWK showed similar inhibitory effects as Ac-RFWK-CMK on CASP1/11-mediated cleavage of recombinant mGSDMD protein (*SI Appendix*, Fig. S12*D*). Collectively, all these in vivo and in vitro experiments suggest that the RFWK inhibitor, with its design based on the RFWK motif, can significantly control the inflammation by blocking inflammasome caspase activity.

## Discussion

In the long-term evolution of the immune system, negative feedback regulatory mechanisms have been developed to control excessive inflammatory response. At present, many studies have reported a wide range of negative feedback mechanisms involved in the regulation of the inflammatory pathway. However, it has not been reported whether inflammasomes have their own negative feedback systems. In this study, we describe a negative feedback regulatory mechanism in which the cleaved N-terminal fragment of GSDMD can inhibit caspase-1/11 activity after inflammasome activation.

Using random structural docking analysis and mutant studies, we identified the specific RFWK motif in the β1-β2 loop of GSDMD N terminus as a critical requirement for the inhibitory effects on caspase-1/11 activity. This region within the β1-β2 loop is masked by the GSDMD-C domain in the autoinhibited full-length structure (37). After GSDMD cleavage, the β1-β2 loop, together with the adjacent α1 and α3 helix, form a positively charged surface patch to bind lipids (31, 32). While a previous study has suggested that the cleavage-site tetrapeptide of GSDMD binds to the catalytic groove of caspase-1 (38), our studies showed that the RFWK residues of the β1-β2 loop were predicted to be more important binding sites for the caspase catalytic center. Accordingly, a recent study found that the cleavage tetrapeptide in GSDMD contributes little to its recognition by caspase-1/11 (28). Thus, our studies expand the new function of the β1-β2 loop and propose insight into a regulatory system in which caspase activation is regulated by a negative feedback system via the N-terminal fragment β1-β2 loop of GSDMD (*SI Appendix*, Fig. S13): Briefly, the autoprocessed caspase subunits produce an exosite to recruit and recognize the GSDMD-C terminus of full-length GSDMD; second, the GSDMD N- and C-terminal domain linker tetrapeptide is engaged into the catalytic pocket of caspases for cleavage. Next, the N-terminal fragment of GSDMD is released and can render its β1-β2 loop, via the RFWK motif, accessible to interaction with the caspase catalytic pocket, thereby controlling caspases activity terminating inflammasome activation.

While we have demonstrated the key role of the RFWK motif of GSDMD N-terminal fragment in manifesting negative feedback regulation of inflammasome activation, the exact structure and nature of interaction between caspases-1/11 enzyme pockets and the β1-β2 loop still needs to be determined by crystal studies. How the dynamic changes of GSDMD N-terminal fragment after cleavage adapt to the binding and inhibition of caspase active sites via its RFWK motif of the β1-β2 loop remains to be investigated.

Inflammasomes play critical roles in many inflammatory disorders. Caspase-1/11 is a key target for the treatment of inflammation-associated disease (39, 40). Based on the RFWK inhibitory motif, we designed an RFWK peptide inhibitor to target caspase-1/11 activity and found the inhibitor substantially suppresses GSDMD cleavage and IL-1β release, as well as LPS-induced sepsis in mice. Thus, the identified RFWK inhibitory motif may provide new valuable clues to develop novel drugs that target caspase-1/11 enzymes and so treat inflammatory diseases. Notably, we found that the Pan-caspase inhibitor z-VAD-FMK, while inhibiting the cleavage of IL-1β and GSDMD, as well as pyroptosis, did not inhibit the production of the caspase-1 P10 fragment but blocked processing to generate a protein larger than 20 kDa in size. This finding contrasts with a previous report that the P10 product autoactivated by caspases is sufficient for cleaving GSDMD and pyroptosis (28). The nature of this larger than 20-kDa fragment awaits further investigation by N-terminal sequencing and mass spectrometry.

Other Gasdermin family molecules, such as GSDME and GSDMB, can be cleaved by caspase-3 and Granzyme A or B (41–43). Whether there is a negative feedback regulation mechanism similar to GSDMD and caspase-1/11 in their activation process is worthy of investigation in future studies. Our study found that the N-terminal of GSDMD plays an alternative role in inhibiting the activation of caspase-1/11 and processing of IL-1β, and so acting to terminate such proinflammatory signaling. This finding may also provide the mechanistic basis to the recent report that showed excessive cell lysis to inhibit the release of IL-1β (32). Thus, the dualist pro- and antiinflammatory effects of the N-terminal fragment of GSDMD can help the body dynamically regulate inflammation and provide that yin-yang balance in immune system to avoid pathological consequences of excessive and uncontrolled inflammation.

## Materials and Methods

Details of experimental procedures and reagents can be found in *SI Appendix, Materials and Methods*. All experiments were performed in accordance with the procedures approved by the Research Ethics Committee of Nanjing Medical University. All mice were kept in a barrier facility, and all animal experiments were conducted in accordance with the procedures approved by the Ethical Review Committee for Laboratory Animal Welfare of Nanjing Medical University.

## Supplementary Material

Supplementary File

## Data Availability

Requests for the protocols, resources and reagents should be directed to and will be fulfilled by author S.Y. (shuoyang@njmu.edu.cn). All other study data are included in the main text and *SI Appendix*.
