# Supplementary material for: The Gasdermin D N-terminal fragment acts as a negative feedback system to inhibit inflammasome-mediated activation of Caspase-1/11
Source: Proc Natl Acad Sci U S A. 2022 Nov 2;119(45):e2210809119. doi: 10.1073/pnas.2210809119 (PMC9659347; doi:10.1073/pnas.2210809119)
Supplement: Supplementary File [file pnas.2210809119.sapp.pdf]

Supporting Information for

The Gasdermin D N-terminal fragment acts as a negative feedback system to inhibit inflammasome-mediated activation of Caspase-1/11

Yingchao Hu[a#], Yuying Jiang[a#], Sheng Li[a,b#], Xiaoqing Ma[c], Min Chen[a], Rui Yang[a], Shuang Wen[a], Paul N Moynagh[d,e], Bingwei Wang[c]*, Gang Hu[c]* and Shuo Yang[a,f]*

[a]Department of Immunology, Key Laboratory of Immunological Environment and Disease, Gusu School, State Key Laboratory of Reproductive Medicine, Jiangsu Key Lab of Cancer Biomarkers, Prevention and Treatment, Collaborative Innovation Center for Personalized Cancer Medicine, Center for Global Health, Nanjing Medical University, Nanjing, China, 211166
[b]Shanghai Key Laboratory of Maternal Fetal Medicine, Clinical and Translational Research Center of Shanghai First Maternity and Infant Hospital, School of Medicine, Tongji University, Shanghai, 200092, China.
[c]Department of Pharmacology, Nanjing University of Chinese Medicine, 138 Xianlin Avenue, Nanjing, China, 210023
[d]Kathleen Lonsdale Institute for Human Health Research, Department of Biology, National University of Ireland Maynooth, Maynooth, Ireland
[e]Wellcome-Wolfson Institute for Experimental Medicine, Queen's University Belfast, Belfast, United Kingdom
[f]Lead contact

# These authors contributed equally.

* Correspondence: shuoyang@njmu.edu.cn (S.Y. Lead contact)
ghu@njmu.edu.cn (G.H.)
bingweiwang@njucm.edu.cn (B.W.W.)

This PDF file includes:

SI Materials and Methods
Figures S1 to S13
SI References

Materials and Methods

Contact for accessing the data, associated protocols, code, and materials

All data are included in the manuscript and/or supporting information. Further information and requests for the protocols, resources and reagents should be directed to and will be fulfilled by the Lead Contact, Shuo Yang (shuoyang@njmu.edu.cn).

Mice

The GSDMD$^{-/-}$ mice were kindly provided by Dr. Feng Shao (National institute of Biological Sciences, Beijing, China). *GSDMD-N*^{LSL} mice were generated by Cyagen Biosciences Inc (Guangzhou, China) using CRISPR-CAS9 technology. Briefly, the donor DNA containing 3×Flag-GSDMD-N fragment with LoxP-flanked stop cassette, the gRNA to mouse ROSA26 gene and CAS9 mRNA were co-injected into the C57BL/6 zygotes to generate targeted knock-in offspring. F0 founder animals identified by PCR and sequence analysis were bred to wild-type mice to test germline transmission and F1 animal generation. F1 heterozygous mice were genotyped by tail genomic PCR and southern blot. *GSDMD-N*^{LSL} mice were crossed with *LysM*-Cre mice (*LysM*-Cre;The Jackson Laboratory) to delete the stop cassette and produce myeloid-specific conditional GSDMD-N fragment expressing mice (*GSDMD-N*^{LSL}; *LysM*-Cre). All mice were kept in a barrier facility, and all animal experiments were conducted in accordance with the procedures approved by the Ethical Review Committee for Laboratory Animal Welfare of Nanjing Medical University.

Cell culture

HEK293T cells and immortalized BMDMs were cultured in Dulbecco's modified Eagle's medium (DMEM) supplemented with 10% (v/v) fetal bovine serum (FBS), 100 μg/ml penicillin G and 100 μg/ml streptomycin. THP-1 cells were grown in RPMI 1640 medium supplemented with 10% (v/v) fetal bovine serum (FBS), 100 μg/ml penicillin G and 100 μg/ml streptomycin. For isolation of primary BMDMs, tibias and femurs of mice were removed by sterile techniques and bone marrow was flushed with fresh medium. BMDMs were cultured in DMEM supplemented with 10% FBS in the presence of 10% L929 conditional medium for 4–6 days at 37 °C in a humidified atmosphere of 5% CO2. Generation of iBMDMs from primary BMDMs was performed as previously described(*1*). Briefly, two rounds of conditioned supernatant collected from the CREJ2 cell line carrying the J2 retrovirus was placed on differentiating macrophage cultures. BMDMs were progressively starved of M-CSF by reducing the L929 supplementation over approximately 1.5 months until cells were able to grow without M-CSF and could be passaged 1:10 every 2-3 days. All experiments involving immortalization of cells were performed in accordance with the procedures approved by the Research Ethics Committee of Nanjing Medical University (1806009-1).

Expression vectors

Complementary DNA for mouse GSDMD and mouse pro-CASP1/11 were synthesized by GenScript (Nanjing, China); cDNA for human GSDMD was synthesized by Sangon Biotech (Shanghai, China); cDNA for mouse pro-IL-1β was amplified from reverse-transcribed cDNA of LPS-stimulated BMDMs. For GSDMD, the full length and C terminal fragment with Flag tag at N terminus, and the N terminal fragment and N terminal fragment mutants (GSDMD-N_{RFWKm}, GSDMD-N_{LLSDm} and GSDMD-$N_{E15K/L193Dm}$) with 3×Flag tag at N terminus were inserted into the lentiviral vectors pLV-EF1a-IRES (Addgene) respectively. For mouse IL-1β, the full length, N terminal and C terminal fragment with Flag tag at C terminus were inserted into the lentiviral vector pLV-EF1a-IRES respectively. HA-tagged full-length mouse GSDMD and Myc-tagged mouse pro-CASP1/11 were cloned into the pcDNA3.1 vector with tag at N terminus. Enzymatically inactive large and small subunit mutants of murine CASP1/11 were inserted into the lentiviral vector pLV-EF1a-IRES (Addgene) with a N-terminal Myc tag. For recombinant expression in E. coli, the full length mGSDMD (mGSDMD-Full), N-terminal mGSDMD (mGSDMD-N) and N-terminal mGSDMD mutants (mGSDMD-N_{RFWKm} and mGSDMD-N_{LLSDm}) were cloned into the pGEX-6P1 vector. Non-tagged p20, p20(C284A) and p10 subunits of mouse CASP1 were cloned into the pET-21a. The catalytically inactive p22 (C254A) and p10 subunits of CASP11 and the p32 fragment of catalytically CASP11 without D285A mutation were gift from Dr. Feng Shao.

Antibodies and reagents

Antibody to CASP1-P20 (AG-20B-0042) and CASP1-P10 (AG-20B-0044) were from Adipogen; Anti-IL-1β (AB-401-NA) was from R&D; Anti-GSDMD (ab209845) , anti-CASP1 (ab207802) were from Abcam; Anti-CASP11 (NB120-10454) and anti-GSDMDC1 (NBP2-33422) were from Novus; Anti-CASP11(17D9-C1354), anti-Flag (F3165) and anti-β-actin (A1978) were from Sigma; anti-HA (16B12-MMS-101P) was from Covance; anti-Myc (2276) was from Cell Signaling Technology; anti-CASP4 was from AiFang biotechnology and Dr. Feng Shao's lab; IRDye 680RD-anti-mouse (926-68070) and IRDye 800CW-anti-rabbit (926-32211) were from LI-COR Biosciences; Anti-mouse-HRP (31340) and anti-goat-HRP (31402) were from Thermo Fisher Scientific; Anti-CD45-FITC (30-F11,11-0451-82), anti-CD8a-PE (53-6.7,12-0081-83), anti-CD11b-APC (M1/70,17-0112-82), anti-IL17-PE(eBio18B7,12-7177-81), anti-IFNγ-APC (XMG1.2,17-7311-82), and FVD eFlour® 506 were from eBioscience; Anti-CD4-APC-Cy7 (GK1.5, 100414) was from Biolegend. LPS (ALX-581-013-L002) and LPS (L-4130) were from Enzolife Sciences and Sigma, respectively; Nigericin (tlrl-nig-5) and poly(dA:dT) (tlrl-patn) were from Invivogen; The pan-caspase inhibitor z-VAD-FMK (HY-16658B) was from MedChemExpress; Ac-RFWK-CMK inhibitor was from KE BIOCHEM. Pertussis toxin (#180) was from List Biological Laboratories; Mycobaterium tuberculosis H37Ra (231141) was from BD; Incomplete Freund's adjuvant (F5506) was from Sigma; MOG35-55 peptide (resdue 35-55, Met-Glu-Val-Gly-Trp-Tyr-Arg-Ser-Pro-Phe-Ser-Arg-Val-Val-His-LeuTyr-Arg-Asn-Gly-Lys) was synthesized by Sangon Biotech (Shanghai).

Immunoprecipitation and immunoblot analysis

HEK293T cells (3×10^5 cells per ml;3ml) grown in six-well plates were transfected with appropriate expression constructs. Cells were then collected in 500 μl lysis buffer (20 mM Tris-HCl, pH 7.4, containing 150 mM NaCl, 0.5% (vol/vol) Igepal, 50 mM NaF, 1 mM Na_3VO_4, 1 mM dithiothreitol, 1 mM phenylmethylsulfonyl fluoride and complete protease-inhibitor 'cocktail'), followed by incubation for 40 min at 4 °C. Cell lysates were initially precleared by centrifugation at 14000 rpm and then followed by incubation for 24 h at 4 °C with anti-Flag magnetic beads (Bimake). Immunoprecipitates were collected by magnetic attraction and the beads were then washed four times with 500 μl lysis buffer (without Na_3VO_4, dithiothreitol, phenylmethylsulfonyl fluoride or protease-inhibitor 'cocktail'). An aliquot (50 μl) of SDS-PAGE sample buffer (62.5 mM Tris-HCl, pH 6.8, 10% (wt/vol) glycerol, 2% (wt/vol) SDS, 0.7 M β-mercaptoethanol and 0.001% (wt/vol) bromophenol blue) was added to the beads. Samples were resolved by SDS-PAGE, transferred to PVDF membranes and analyzed by immunoblot with appropriate antibodies.

Lentiviral transduction of iBMDMs

The full length mGSDMD (mGSDMD-Full), N-terminal mGSDMD (mGSDMD-N), C-terminal mGSDMD (mGSDMD-C) and N-terminal mGSDMD mutants (mGSDMD-N_{RFWKm} and mGSDMD-N_{LLSDm}) and the full length hGSDMD (hGSDMD-Full), N-terminal hGSDMD (hGSDMD-N), C-terminal hGSDMD (hGSDMD-C) were cloned into the lentiviral vector pLV-EF1a-IRES (Addgene). Constructs were transfected into HEK293T cells together with packaging and envelope plasmids (psPAX2 and pMD2.G). Plv lentivirus from the medium supernatants were harvested at 48 h and 72 h by centrifugation and filtration through a 0.45 mm filter. Immortalized BMDM cells and THP-1 cells were seeded at 1.5 $\times10^5$ cells/well in 12-well plates and infected the next day with viral supernatants containing 8μg/ml polybrene. Infection was repeated 36 h later. Cells stably expressing exogenous protein was selected by the addition of proper antibiotic and confirmed by immunoblot.

GSDMD Knockout in THP-1 cells using CRISPR-Cas9

Sequences targeting human GSDMD (5'-CACCGTCTCCGGACTACCCGCTCAA-3' and 5'-CACCGACCAGCCTGCAGAGCTCCAC-3') were cloned into lentiCRISPRv2GFP vector (Addgene) containing Cas9 fused to G418 resistance gene. The plasmids were co-transfected into HEK293T cells with packing plasmids psPAX2 and pMD2. THP-1 cells were transduced with virus from the HEK293T cells and selected with G418 (400μg/ml). Clonal cells were isolated and the loss of GSDMD was verified by Western blot.

Inflammasome activation

Primary BMDMs or immortalized BMDMs or THP-1 cells were seeded in 12-well culture dishes (1.5 $\times 10^6$ cells per well) and cultured overnight. The cells were primed for 4 h with 200ng/ml LPS, and then stimulated with 10μM nigericin for 1 h, or transfected with 2mg/ml poly (dA:dT) for 16 h, or infected with log-phase S.typhimurium (ATCC 14028 ; MOI=30) or Escherichia coli (ATCC 11775 ; MOI=50) for 16h. For electroporation, 2 μg LPS in buffer R was electroporated into BMDMs or iBMDMs under pulse voltage 1400 V, pulse width 10 ms, and pulse number 2, using the Neon Transfection System (Thermo Fisher Scientific) according to the manufacturer's protocol. For detection of inflammasome activation by immunoblot analysis, cells were lysed by the addition of 10 × cell lysis buffer (10% Nonidet P-40 and 10 × protease inhibitors) to the well containing both cells and culture media (i.e., without removing the condition medium) at a final concentration of 1% Nonidet P-40 and protease inhibitors. Cells were then lysed (in the presence of culture medium) on ice for 30 min and centrifuged at 16,000 r.p.m. Loading buffer (4×) was then added into the lysate. Samples were resolved by SDS-PAGE, transferred to nitrocellulose membranes and analyzed by immunoblot with appropriate antibodies.

Cell death assays

Cell death was determined by the lactate dehydrogenase release assay using the Pierce LDH Cytotoxicity Assay Kit (Thermo) according to the manufacturer's instructions. To assess cell permeability, cells were cultured in Opti-MEM containing 10nM SYTOX Green and Hochest. Fluorescence for SYTOX (excitation and emission maximum at 504nm and 523nm) and Hochest (excitation and emission maximum at 360nm and 450nm) were continuously recorded for 60 min after nigericin treatment at 5 min intervals using a BioTek Synergy plate reader.

ELISA

Primary BMDMs were seeded (1.5×10^6 cells per well) in 12-well plates and were grown for 24 h. Cells were then stimulated as indicated in figure legends. Conditional media were collected and measured for the level of TNF-α (DY410) and IL-1β (DY401) according to manufacturer's instructions (R&D System). The serum of EAE and sepsis model mice were collected and the level of IL-1β (DY401) was measured by sandwich ELISA (R&D System).

FAM-FLICA staining

BMDMs or iBMDMs (1.5×10^6 cells per well in a 12-well plate) were treated as indicated for inflammasome activation, and then washed by PBS and resuspended in 300μl PBS. Cells were incubated with 10μl 30 × FAM-YVAD-FLICA (ImmunoChemistry Technologies) at 37 °C for 30 min, protected from light. The assay was performed with Flow cytometer and data was analyzed using FlowJo software.

Recombinant Protein Expression and Purification

E. coli BL21(DE3) cells harboring mGSDMD constructs were induced overnight at 16 °C with 0.5 mM Isopropyl-B-D-thiogalactopyranoside (IPTG). GST-tagged proteins were firstly purified with GSTrap™ FF (GE Healthcare). The eluted protein was purified by Superdex 200 10/300 GL column (GE Healthcare Life Sciences). For recombinant expression of non-covalent complex of mCASP11 C/A, a plasmid combination of a large (p22-C/A) and a small subunit (p10) were co-transformed into E. coli BL21 (DE3). The Fusion proteins with 6×His-SUMO tag were affinity-purified by Ni-Sepharose beads (GE Healthcare Life Sciences). The 6×His-SUMO tag was removed by digestion with SUMO protease at 4°C overnight. The untagged proteins were further purified by Superdex 200 10/300 GL column (GE Healthcare Life Sciences). The p22/p10 complex of catalytically active CASP11 was purified by Ni-Sepharose beads in buffer B containing 20 mM Tris-HCl (pH 8.0), 300 mM NaCl, 20 mM imidazole, and 10 mM β-mercaptoethanol, and eluted with buffer B containing 300 mM imidazole. The eluted proteins were further purified by Superdex 200 10/300 GL column. Expression and purification of the p20/p10 complex of catalytically active and inactive mCASP1 were performed as previously reported refolding method. In brief, non-tagged p20 and p10 subunits proteins were induced with 1 mM IPTG for 4 h at 37 °C. Bacterial pellets containing the inclusion

body were collected, washed twice with buffer C containing 50 mM Tris-HCl (pH 8.0), 300 mM NaCl, and 1 M guanidinium hydrochloride (GdnCl), and then solubilized in buffer D containing 6.5 M GdnCl, 25 mM Tris-HCl (pH 7.5), 5 mM EDTA, and 100 mM DTT. Equal molar amounts of each subunit were mixed to a total volume of 5 mL and quickly diluted into 250 mL of refolding buffer containing 100 mM HEPES (pH 8.0), 100 mM NaCl, 100 mM sodium malonate, 20% sucrose, 0.5 M NDSB-201, and 10 mM DTT. The refolded proteins were concentrated and further purified by Superdex 200 10/300 GL column. All purified proteins were stored in buffer G containing 25 mM Tris-HCl (pH 8.0), 150 mM NaCl and 5 mM DTT.

Thermal shift Assay
Thermal shift assay was performed utilizing the melt capability of Applied Biosystems real-time PCR systems with thermal shift dye kit (cat # 4461146, Thermo Fisher Scientific). 10μM Ac-RFWK-CMK inhibitor was incubated with 3μg purified CASP1/11 complex in a 20μl mixture, and then the mixture was heated in a temperature gradient of 25-95°C with 1% ramp rate. Fluorescence data was fitted with the Boltzmann equation to obtain melting temperature using GraphPadPrism (GraphPad Software, San Diego, CA).

In vitro GSDMD cleavage by recombinant CASP1/11
To examine cleavage of GSDMD by the p20/p10 tetramers of active caspase, 5μg substrate proteins were incubated with 0.1μg mCASP1/11 in the presence of 0.2μg mGSDMD-N or mGSDMD-N mutants ($mGSDMD\text{-}N_{RFWKm}$ and $mGSDMD\text{-}N_{LLSDm}$) protein or Ac-RFWK-CMK inhibitor at 37 °C for 60 min. SDS loading buffer was then added to the reaction mixture followed by boiling at 37 °C for 5 min. The samples were analyzed by SDS-PAGE and Coomassie brilliant blue staining.

GST Pull-Down
Immobilized GST-tagged mGSDMD-N or mGSDMD-N mutants ($mGSDMD\text{-}N_{RFWKm}$ and $mGSDMD\text{-}N_{LLSDm}$) protein was incubated with mCASP1/11 (C/A) at a molar ratio of 1:3 in a buffer containing 20 mM Tris-HCl pH 8.0, 300 mM NaCl and 1 mM DTT at 4 °C for 24 h. The GST resin was washed four times with the same buffer, and the bound protein was eluted using 20 mM reduced L-Glutathione. The input and eluted samples from pull-down were analyzed with SDS-PAGE.

Molecular modelling and docking methods
The 3D structure of full length murine GSDMD (6N9N), full length murine GSDMA3 (5B5R), murine GSDMA3 pore form (6CB8), human GSDMD pore form (6VFE), human CASP1 (C285A)-p20/p10 (6KN0) and CASP11 (C254A)-p22/p10 (6KMU) were obtained from the Protein Data Bank. The activating pore conformation of murine GSDMD-N fragment with flexible cleavage linker ($mGSDMD\text{-}N_a$) was constructed by using program Modeller based on the pore structure of murine GSDMA3 (PDB: 6CB8) and human GSDMD (6VFE). And the inactivating conformation of murine GSDMD-N fragment in full-length mGSDMD with cleavage linker ($mGSDMD\text{-}N_i$) was generated with program Modeller based on the structure of full-length murine GSDMD (PDB: 6N9N). The structural model of murine CASP1 (p20/p10) was generated based on the structure of human CASP1 (C285A)-p20/p10 (PDB: 6KN0) by homology-modeling (SWISS-MODEL). All the modelled protein structures were validated in Ramachandran plot by Schrödinger analysis. The prepared structures of the mGSDMD-N (mGSDMD-Na and mGSDMD-Ni) and mCASP1/11 were submitted to ZDOCK SERVER without residues selected for induced-fit random docking to predict the binding modes. The random docking results were also verified using Schrödinger analysis.

Induction and assessment of EAE
To induce EAE model, MOG35-55 peptide (200 μg per mouse) emulsified with complete Freund's adjuvant (CFA, 50 μl per mouse, including 4 mg/ml M. tuberculosis H37Ra) and incomplete Freund's adjuvant (IFA, 50μl per mouse) was subcutaneously injected on day 0. Pertussis toxin (250 ng per mouse) was applied intravenously on day 0 and 2 after immunization. Mice were assessed daily for clinical signs of EAE in a blinded fashion. EAE score was evaluated as follows: 0.5, partial tail paralysis; 1, tail paralysis; 1.5, reversible corrective reflex impairment; 2, corrective

reflex impairment; 2.5, one hindlimb paralysis; 3, both hindlimbs paralysis; 3.5, both hindlimbs paralysis and one forelimb paralysis; 4, hindlimb and forelimb paralysis; and 5, death.

Isolation of CNS immune cells and FACS analysis

For preparation of immune cells, brains and spinal cords from MOG35–55-immunized mice were excised and digested at 37°C with DNase I (10 U/ml; Roche) and collagenase type IV (0.5mg/ml; Sigma) in RPMI 1640 under agitation (200 rpm) conditions for 60 min. Single cell suspensions were obtained by grinding through 70 μm cell strainer. Subsequently, homogeneous cell suspensions were centrifuged over the Percoll density gradient (GE Healthcare) and separated by collecting the interface fractions between 37% and 70% Percoll. Mononuclear cells were isolated from the interface. After intensive washing, single cell suspensions were stained with FVD eFlour® 506, anti-CD45, anti-CD4, anti-CD8 and anti-CD11b for FACS analysis. For intracellular cytokine staining, cells were stimulated with phorbol 12-myristate 13-acetate (Multi Sciences), ionomycin (Multi Sciences) and brefeldin A (Invitrogen) for 4 h of culture. Cells were fixed and permeabilized with Intracellular Fixation & Permeabilization Buffer Set (eBioscience) and then subjected to cytokine staining flow cytometry analysis. All flow cytometry was performed on an Attune NxT flow cytometer (Thermo fisher) and data was analyzed by Flow Jo 10.0.7 software.

Drug administration and LPS-induced sepsis in mice

8 to 12-week-old age wild-type mice were treated with Ac-RFWK-CMK inhibitor (25 mg/kg) or vehicle (ctrl) by intraperitoneal injection. After 4h, sepsis was induced in C57BL/6 mice by intraperitoneal injection of LPS (Escherichia coli O111:B4) (25mg/kg). Peritoneal cells were collected 6 h after LPS challenge by rinsing the peritoneal cavity with ice cold PBS containing 3% FBS. To measure cytokines, blood samples were collected 6 h post LPS challenge and allowed to clot at room temperature. Sera was obtained after centrifugation at 2,000g for 10 min and analyzed for inflammatory cytokines by enzyme linked immunosorbent assay (ELISA).

Statistical Analysis

Data are presented as the mean ± standard error of the mean (SEM). Samples were analyzed using unpaired t test for two groups and ANOVA for multiple groups. Log-rank (Mantel-Cox) test for Survival curves. In all cases, a P value of less than 0.05 was considered statistically significant.

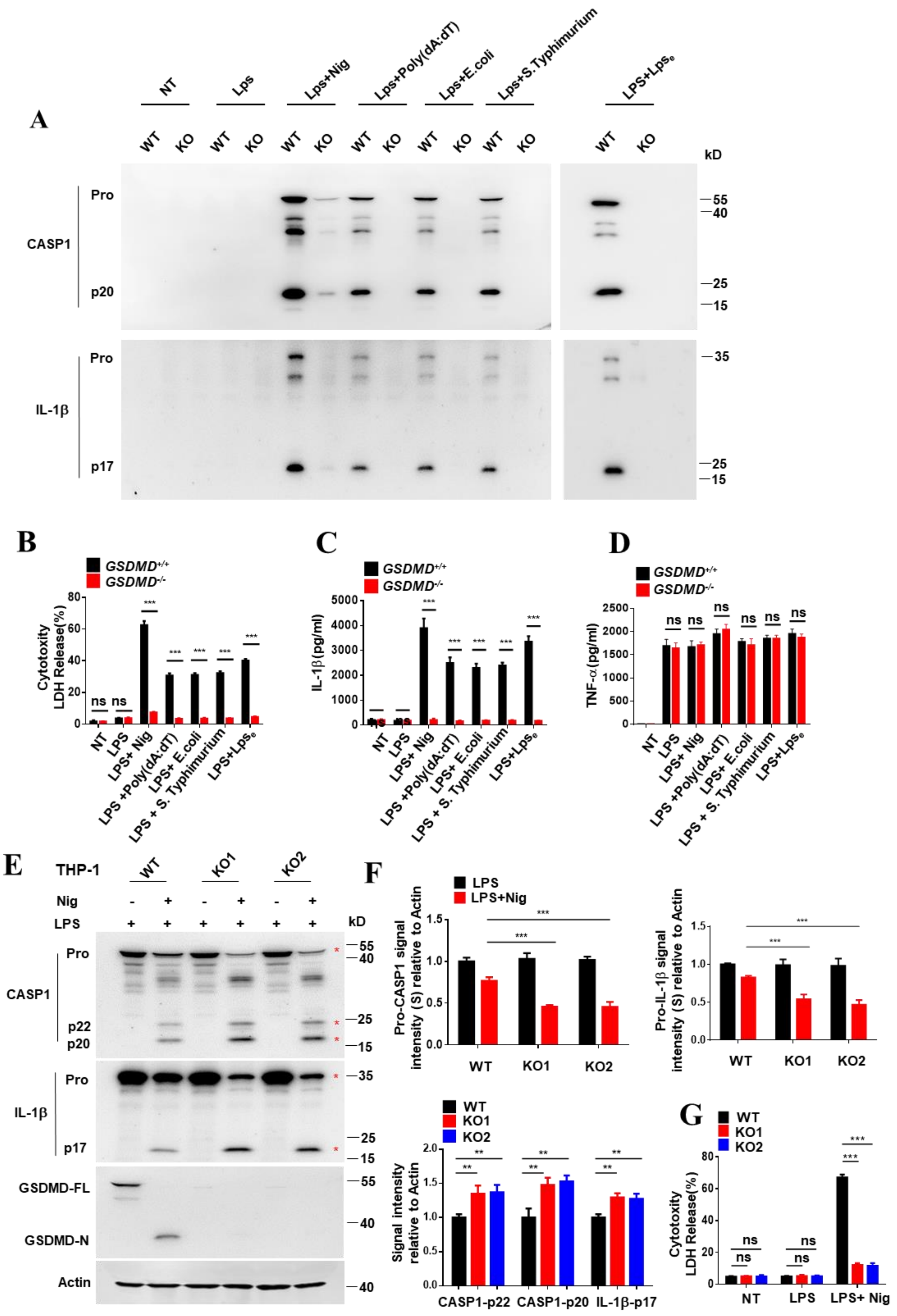
A
NT
Lps
Lps+Nig
Lps+Poly(dA:dT)
Lps+E.coli
Lps+S.Typhimurium
LPS+Lpse
WT KO
kD
CASP1
Pro
p20
IL-1β
p17
B
C
D
GSDMD+/+
GSDMD-/-
Cytotoxicity LDH Release(%)
IL-1β(pg/ml)
TNF-α(pg/ml)
NT
LPS
LPS+ Nig
LPS +Poly(dA:dT)
LPS+ E.coli
LPS + S. Typhimurium
LPS+Lpse
ns
***
E
THP-1
WT
KO1
KO2
Nig
LPS
CASP1
p22
GSDMD-FL
GSDMD-N
Actin
F
LPS
LPS+Nig
Pro-CASP1 signal intensity (S) relative to Actin
Pro-IL-1β signal intensity (S) relative to Actin
Signal intensity relative to Actin
CASP1-p22
CASP1-p20
IL-1β-p17
G
**

Fig. S1. GSDMD deficiency impairs pyroptosis and the release of CASP1 and IL-1β in supernatant after inflammasome activation.

(A) Immunoblot analysis of supernatants from primary wild-type and *GSDMD*$^{-/-}$ BMDMs after canonical and non-canonical inflammasome stimulation.
(B) LDH released from wild-type and *GSDMD*$^{-/-}$ BMDMs upon inflammasome activation as indicated.
(C and D) ELISA analysis of IL-1β and TNF-α in the supernatants from primary wild-type and *GSDMD*$^{-/-}$ BMDMs in response to inflammasome activation.
(E) Immunoblot analysis of pooled cell extracts and supernatants from wild-type and *GSDMD*$^{-/-}$ THP-1 cells primed with LPS (200ng/ml) for 4h followed by Nigericin (Nig.; 10μM) stimulation for 1 h.
(F) Relative quantification analysis based on grayscale values of (E). * indicates the bands used for grayscale analysis.
(G) LDH released from wild-type and *GSDMD*$^{-/-}$ THP-1 cells upon NLRP3 inflammasome activation as indicated.
Data are representative of three independent experiments (A and E) or are pooled from three independent experiments (B, C, D，F and G). **$p < 0.01$, ***$p < 0.001$, ns, not significant. Two-way ANOVA with Sidak’s multiple comparisons test for (B, C, D, F and G).

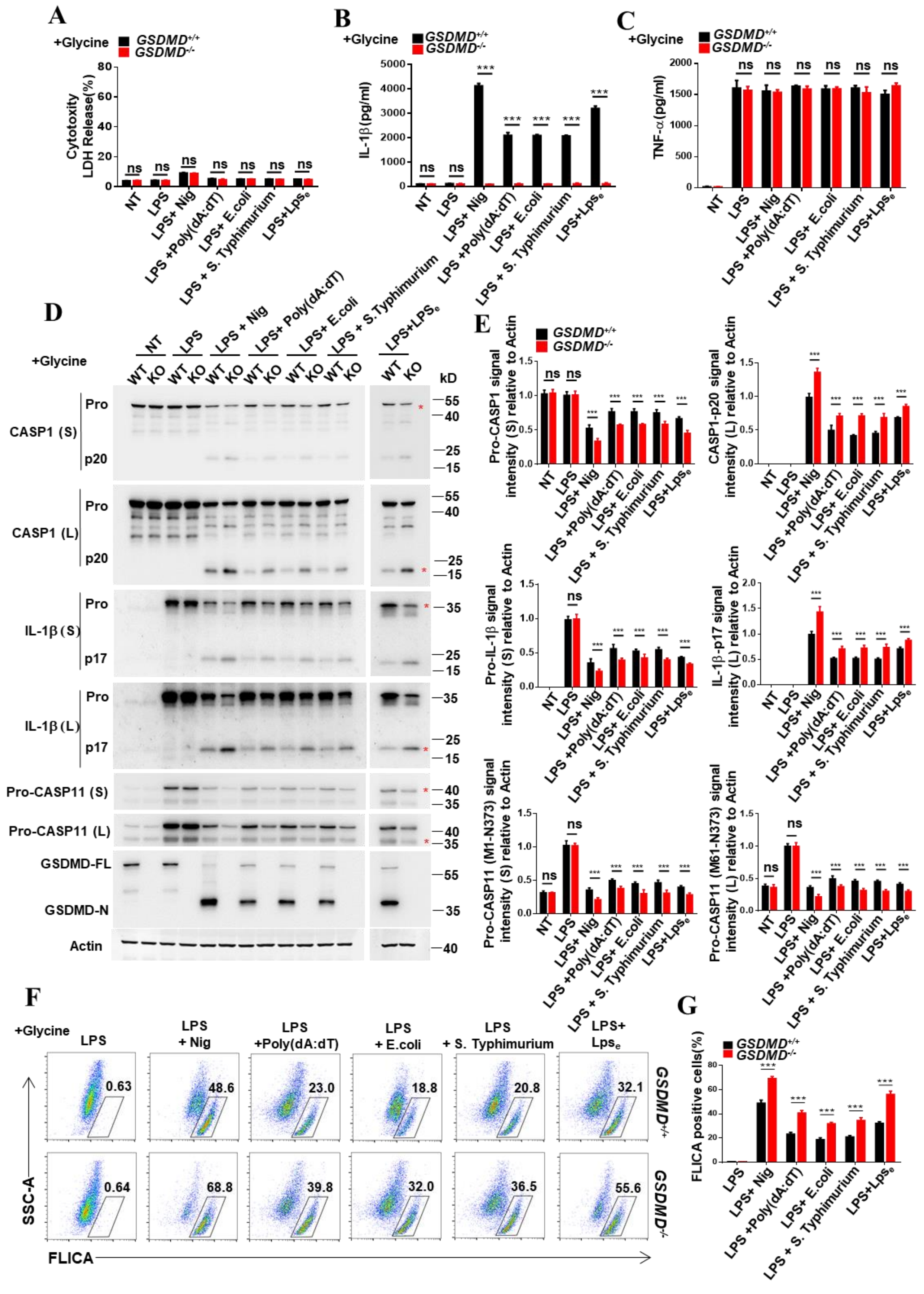

A
+Glycine GSDMD+/+ GSDMD-/-
Cytotoxity LDH Release(%)
B
IL-1β(pg/ml)
C
TNF-α(pg/ml)
NT
LPS
LPS+ Nig
LPS +Poly(dA:dT)
LPS+ E.coli
LPS + S. Typhimurium
LPS+Lpse
D
CASP1 (S)
CASP1 (L)
IL-1β (S)
IL-1β (L)
Pro-CASP11 (S)
Pro-CASP11 (L)
GSDMD-FL
GSDMD-N
Actin
Pro
p20
p17
WT KO
kD
E
Pro-CASP1 signal intensity (S) relative to Actin
CASP1-p20 signal intensity (L) relative to Actin
Pro-IL-1β signal intensity (S) relative to Actin
IL-1β-p17 signal intensity (L) relative to Actin
Pro-CASP11 (M1-N373) signal intensity (S) relative to Actin
Pro-CASP11 (M61-N373) signal intensity (L) relative to Actin
F
LPS
LPS + Nig
LPS +Poly(dA:dT)
LPS + E.coli
LPS + S. Typhimurium
LPS+ Lpse
0.63
48.6
23.0
18.8
20.8
32.1
0.64
68.8
39.8
32.0
36.5
55.6
SSC-A
FLICA
G
FLICA positive cells(%)

Fig. S2. GSDMD deficiency exacerbates inflammasome activation in the presence of Glycine

(A) LDH released from wild-type and *GSDMD*$^{-/-}$ BMDMs treated with canonical and non-canonical inflammasome stimuli as indicated in the presence of glycine (5mM).

(B and C) ELISA analysis of IL-1β and TNF-α in the supernatants from primary wild-type and *GSDMD*$^{-/-}$ BMDMs upon inflammasome activation in the presence of glycine (5mM).

(D) Immunoblot analysis of pooled cell extracts and supernatants from primary wild-type and *GSDMD*$^{-/-}$ BMDMs after canonical and non-canonical inflammasome stimulation in the presence of glycine (5mM).

(E) Relative quantification analysis based on grayscale values of (D). Referring to Actin, the signal intensity of pro-CASP1, pro-IL-1β and pro-CASP11(M1-N373) were calculated by the gray value of shorter exposure; the CASP1, IL-1β and pro-CASP11(M61-N373) were calculated by the gray value of longer exposure. * indicates the bands used for grayscale analysis.

(F and G) Flow-cytometric analysis of CASP1/11 activation probed by FLICA-positive staining in wild-type and *GSDMD*$^{-/-}$ BMDMs during canonical or non-canonical inflammasome activation as indicated in the presence of glycine (5mM).

Data are pooled from three independent experiments (A, B, C, E and G) or are representative of three independent experiments (D and F). Error bars show means ± SEM. ***p <0.001, ns, not significant. Two-way ANOVA with Sidak’s multiple comparisons test for (A, B, C, E and G).

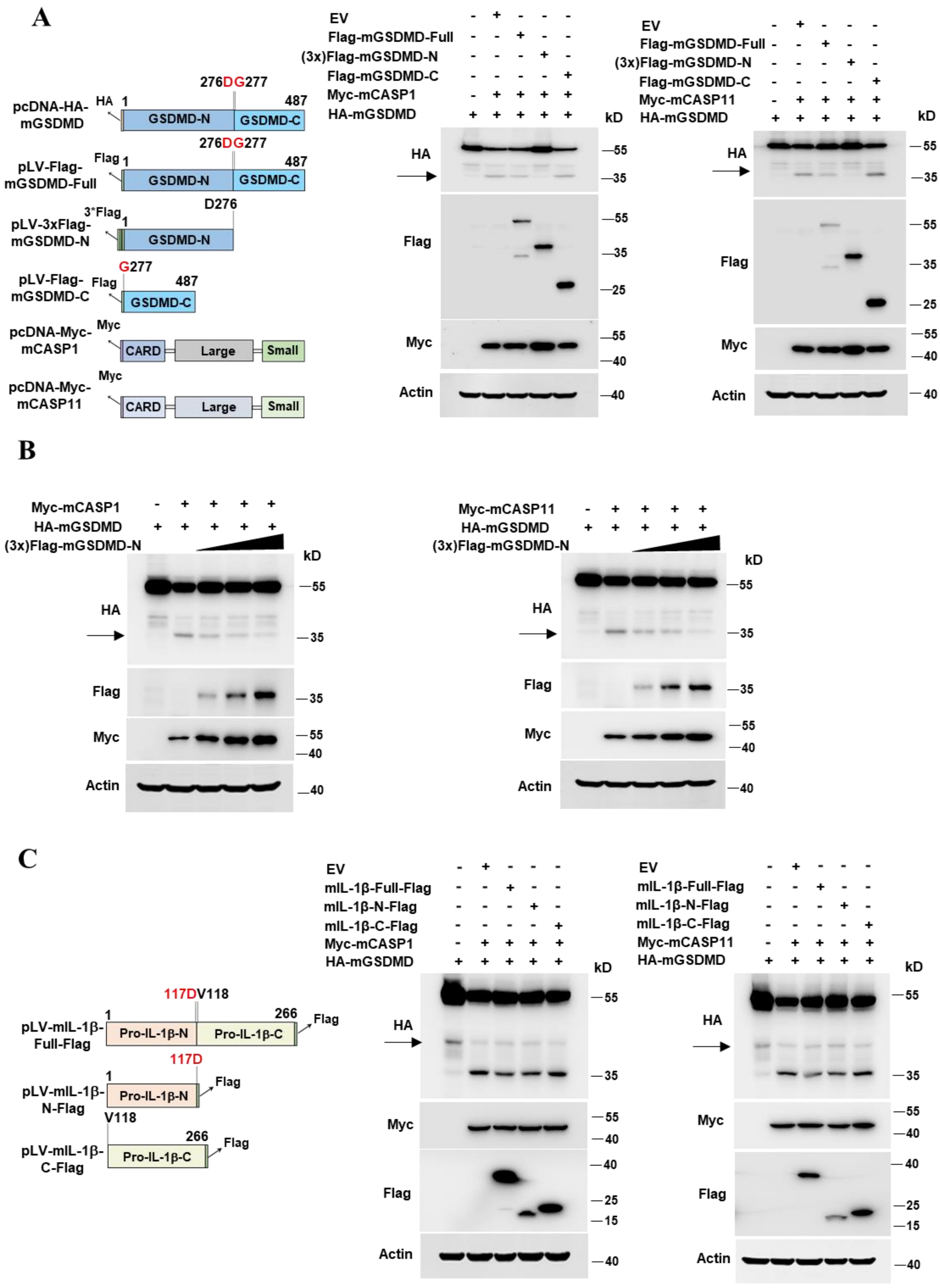
A
pcDNA-HA-mGSDMD
HA 1 276DG277 487
GSDMD-N GSDMD-C
pLV-Flag-mGSDMD-Full
Flag 1 276DG277 487
pLV-3xFlag-mGSDMD-N
3*Flag 1 D276
pLV-Flag-mGSDMD-C
Flag G277 487
GSDMD-C
pcDNA-Myc-mCASP1
Myc CARD Large Small
pcDNA-Myc-mCASP11
Myc CARD Large Small
EV
Flag-mGSDMD-Full
(3x)Flag-mGSDMD-N
Flag-mGSDMD-C
Myc-mCASP1
HA-mGSDMD
kD
HA
Flag
Myc
Actin
55
35
25
40
EV
Flag-mGSDMD-Full
(3x)Flag-mGSDMD-N
Flag-mGSDMD-C
Myc-mCASP11
HA-mGSDMD
kD
HA
Flag
Myc
Actin
B
Myc-mCASP1
HA-mGSDMD
(3x)Flag-mGSDMD-N
kD
HA
Flag
Myc
Actin
Myc-mCASP11
HA-mGSDMD
(3x)Flag-mGSDMD-N
kD
HA
Flag
Myc
Actin
C
pLV-mIL-1β-Full-Flag
1 117DV118 266 Flag
Pro-IL-1β-N Pro-IL-1β-C
pLV-mIL-1β-N-Flag
1 117D Flag
Pro-IL-1β-N
pLV-mIL-1β-C-Flag
V118 266 Flag
Pro-IL-1β-C
EV
mIL-1β-Full-Flag
mIL-1β-N-Flag
mIL-1β-C-Flag
Myc-mCASP1
HA-mGSDMD
kD
HA
Myc
Flag
Actin
15
EV
mIL-1β-Full-Flag
mIL-1β-N-Flag
mIL-1β-C-Flag
Myc-mCASP11
HA-mGSDMD
kD
HA
Myc
Flag
Actin

Fig. S3. The N-terminal fragment of GSDMD inhibits CASP1/11 activity

(A) Left, domain architecture of Flag-tagged full length (mGSDMD-Full), N-terminal (mGSDMD-N) and C-terminal (mGSDMD-C) mouse GSDMD, Myc-tagged mouse CASP1/11(mCASP1/11) and HA-tagged full-length mouse GSDMD (HA-mGSDMD). Right and middle, immunoblot analysis of cleavage of HA-tagged mouse GSDMD (HA-mGSDMD) by mCASP1/11 in the presence of Flag-tagged full length (mGSDMD-Full), N-terminal (mGSDMD-N) and C-terminal (mGSDMD-C) mouse GSDMD in HEK293T cells.

(B) Immunoblot analysis of cleavage of HA-tagged mouse GSDMD by CASP1/11 in the presence of Flag-tagged mouse GSDMD-N terminus with different constructs gradient.

(C) Left, domain architecture of Flag-tagged full length (mIL-1β-Full), N-terminal (mIL-1β-N) and C-terminal (mIL-1β-C) mouse IL-1β. Right and middle, immunoblot analysis of cleavage of HA-tagged mouse GSDMD by CASP1/11 in the presence of Flag-tagged full length (mIL-1β-Full), N-terminal (mIL-1β-N) and C-terminal (mIL-1β-C) mouse IL-1β in HEK293T cells.

Data are representative of three independent experiments (A, B and C).

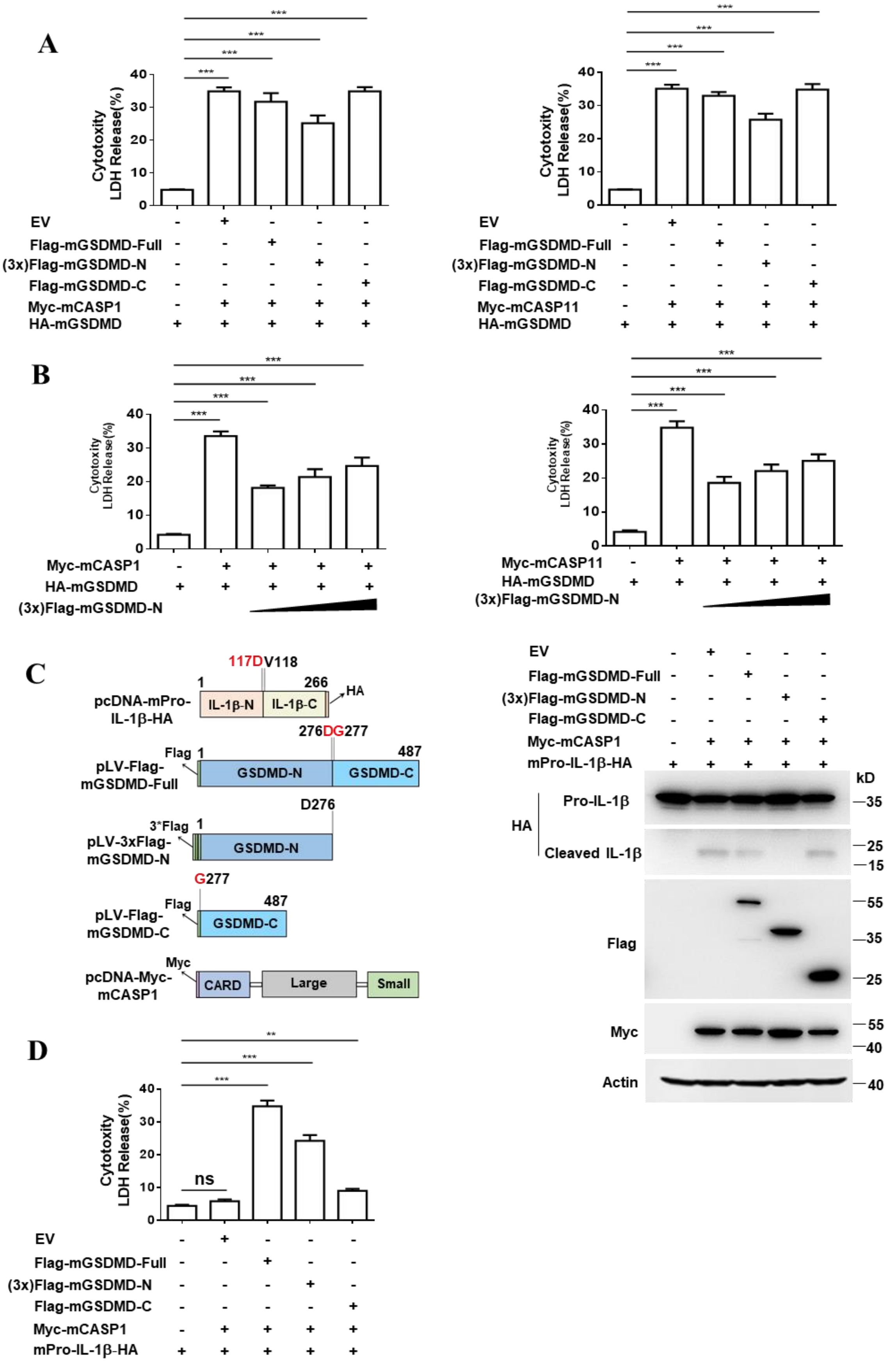

A
Cytotoxity LDH Release(%)
EV
Flag-mGSDMD-Full
(3x)Flag-mGSDMD-N
Flag-mGSDMD-C
Myc-mCASP1
HA-mGSDMD
Myc-mCASP11
B
(3x)Flag-mGSDMD-N
C
117DV118
pcDNA-mPro-IL-1β-HA
IL-1β-N
IL-1β-C
HA
276DG277
pLV-Flag-mGSDMD-Full
GSDMD-N
GSDMD-C
D276
3*Flag
pLV-3xFlag-mGSDMD-N
G277
pLV-Flag-mGSDMD-C
Myc
pcDNA-Myc-mCASP1
CARD
Large
Small
mPro-IL-1β-HA
kD
Pro-IL-1β
Cleaved IL-1β
Flag
Actin
D
ns

Fig. S4. Analysis of cell death and cleavage of pro-IL-1β in cells expressing CASP1/11 and mutants of GSDMD.

(A) LDH released from HEK293T cells co-transfected with HA-mGSDMD, Myc-mCASP1(left panel)/11(right panel) in the presence of Flag-tagged mGSDMD-Full, mGSDMD-N or mGSDMD-C.

(B) LDH released from HEK293T cells co-transfected with HA-mGSDMD, Myc-mCASP1(left panel)/11(right panel) and Flag-tagged mGSDMD-N with different constructs gradient.

(C) Left, domain architecture of Flag-tagged full length (mGSDMD-Full), N-terminal (mGSDMD-N) and C-terminal (mGSDMD-C) mouse GSDMD, Myc-tagged mouse CASP1(mCASP1) and HA-tagged pro-IL-1β (mIL-1β-Full-HA). Right, immunoblot analysis of cleavage of HA-tagged pro-IL-1β (mIL-1β-Full-HA) by mCASP1 in the presence of Flag-tagged mGSDMD-Full, mGSDMD-N or mGSDMD-C in HEK293T cells.

(D) LDH released from HEK293T cells co-transfected with mIL-1β-Full-HA, Myc-mCASP1 in the presence of Flag-tagged mGSDMD-Full, mGSDMD-N or mGSDMD-C.

Data are pooled from three independent experiments (A, B and D) or are representative of three independent experiments (C). Error bars show means ± SEM. ** $p < 0.01$, ***$p < 0.001$, ns, not significant. Two-way ANOVA with Sidak's multiple comparisons test for (A, B and D).

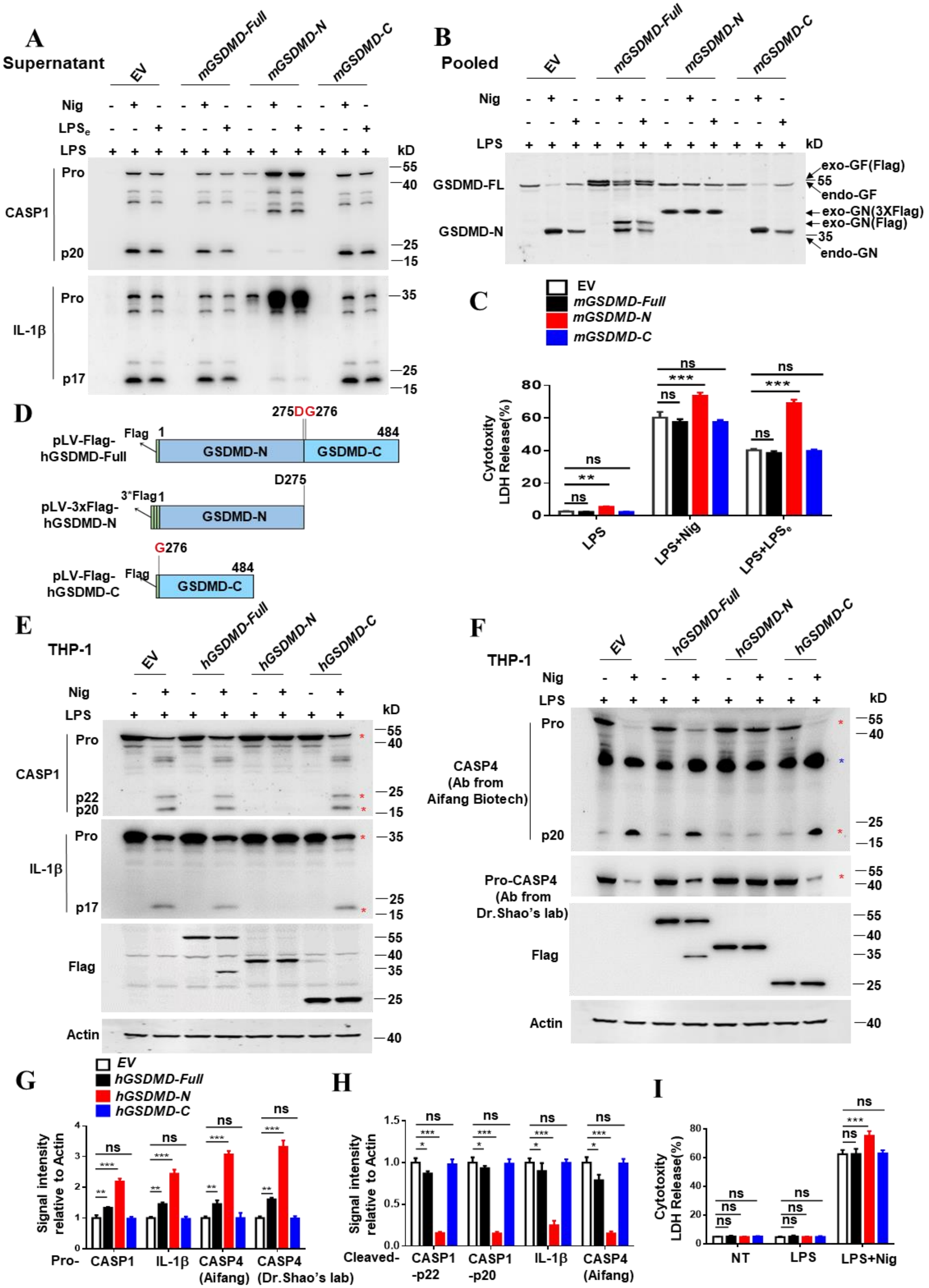
A
Supernatant
EV
mGSDMD-Full
mGSDMD-N
mGSDMD-C
Nig
LPSe
LPS
kD
CASP1
Pro
p20
IL-1β
p17
B
Pooled
GSDMD-FL
GSDMD-N
exo-GF(Flag)
endo-GF
exo-GN(3XFlag)
exo-GN(Flag)
endo-GN
C
Cytotoxity
LDH Release(%)
LPS
LPS+Nig
LPS+LPSe
D
pLV-Flag-
hGSDMD-Full
pLV-3xFlag-
hGSDMD-N
pLV-Flag-
hGSDMD-C
275DG276
D275
G276
484
GSDMD-N
GSDMD-C
E
THP-1
EV
hGSDMD-Full
hGSDMD-N
hGSDMD-C
p22
Flag
Actin
F
CASP4
(Ab from
Aifang Biotech)
Pro-CASP4
(Ab from
Dr.Shao's lab)
G
Signal intensity
relative to Actin
Pro-
CASP4
(Aifang)
(Dr.Shao's lab)
H
Cleaved-
CASP1
-p22
-p20
I
NT
LPS+Nig

Fig. S5. Inflammasome-mediated release of LDH, CASP1 and IL-1β, and CASP1/4 and IL-1β processing in iBMDM and THP-1 cells stably expressing GSDMD-Full, GSDMD-N and GSDMD-C

(A) Immunoblot analysis of supernatants from iBMDMs stably expressing mGSDMD-Full, mGSDMD-N and mGSDMD-C during canonical (LPS-Nig) and non-canonical (LPS electroporation) inflammasome activation.

(B) Immunoblot analysis of endogenous and exogenous GSDMD expression by GSDMD antibody in pooled supernatants and cell extracts from iBMDMs stably expressing mGSDMD-Full, mGSDMD-N and mGSDMD-C following canonical (LPS-Nig, Nig for 1 h) and non-canonical (LPS electroporation for 6h) inflammasome activation. Endo-GF, endogenous full-length GSDMD; endo-GN, endogenous GSDMD N-terminal fragments; exo-GF(Flag), exogenous full length GSDMD with Flag tag; exo-GN(Flag), exogenous GSDMD N-terminal fragments with Flag tag; exo-GN(3xFlag), exogenous GSDMD N-terminal fragments with 3xFlag tag.

(C) LDH released from iBMDMs stably expressing mGSDMD-Full, mGSDMD-N and mGSDMD-C upon inflammasome activation as indicated.

(D) Plasmids of Flag-tagged full length (hGSDMD-Full), N-terminal (hGSDMD-N) and C-terminal (hGSDMD-C) human GSDMD for stable expression in THP-1 cells.

(E and F) Immunoblot analysis of pooled cell extracts and supernatants from THP-1 cells stably expressing hGSDMD-Full, hGSDMD-N and hGSDMD-C during LPS-Nig (Nig for 1h) triggered processing of pro-CASP1/4 and pro-IL-1β. * indicates non-specific bands.

(G and H) Relative quantification analysis based on grayscale values of (E and F). * indicates the bands used for grayscale analysis.

(I) LDH released from THP-1 cells stably expressing hGSDMD-Full, hGSDMD-N and hGSDMD-C after NLRP3 inflammasome activation.

Data are representative of three independent experiments (A and E-F) or are representative of two independent experiments (B) or are pooled from three independent experiments (C and G-I). $*p < 0.05$, $**p < 0.01$, $***p < 0.001$, ns, not significant. Two-way ANOVA with Sidak's multiple comparisons test for (C and G-I).

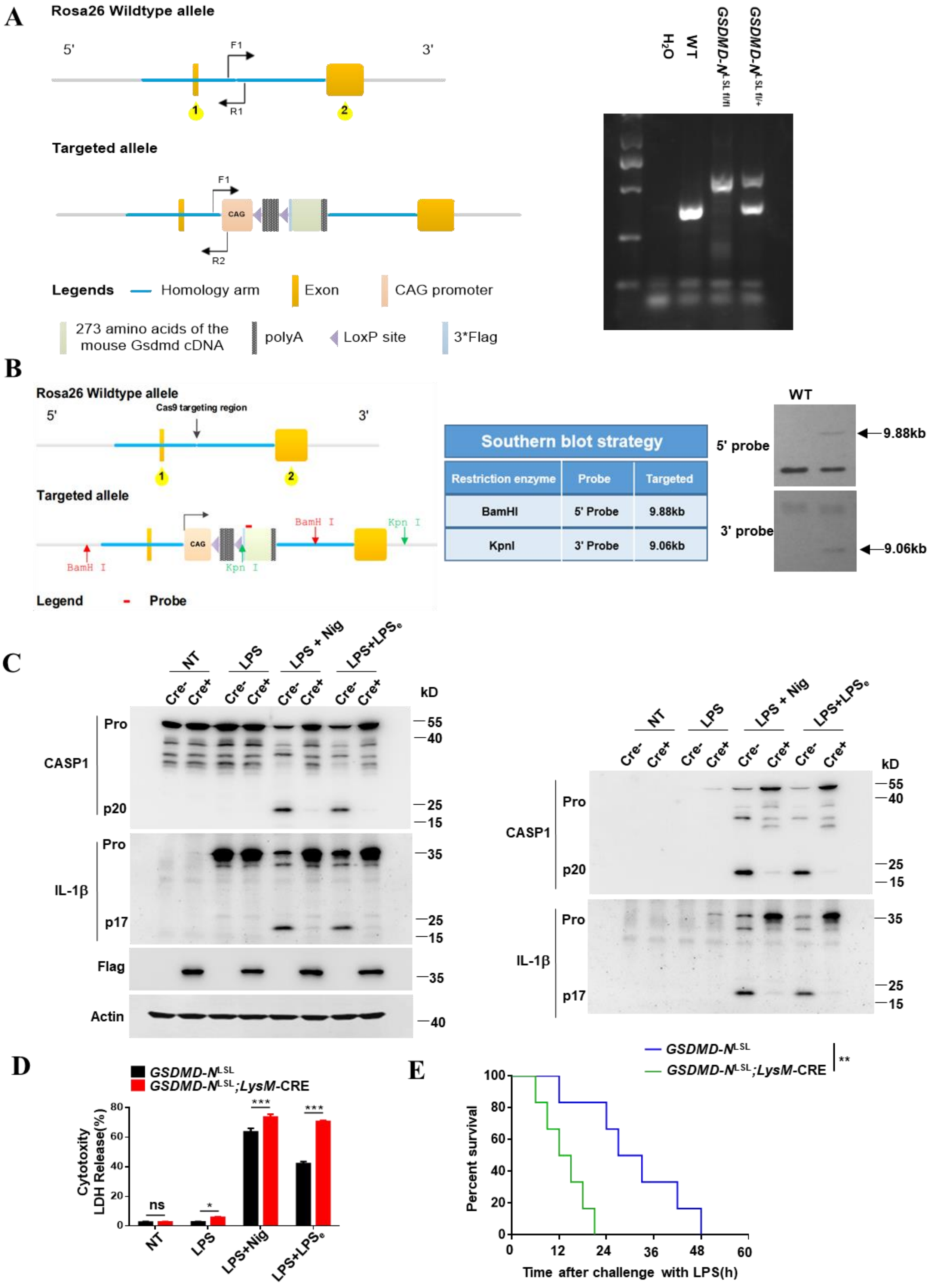
A
Rosa26 Wildtype allele
5'
3'
F1
R1
1
2
Targeted allele
F1
CAG
R2
Legends
Homology arm
Exon
CAG promoter
273 amino acids of the mouse Gsdmd cDNA
polyA
LoxP site
3*Flag
H2O
WT
GSDMD-N LSL fl/fl
GSDMD-N LSL fl/+
B
Rosa26 Wildtype allele
5'
Cas9 targeting region
3'
1
2
Targeted allele
BamH I
Kpn I
CAG
Legend
Probe
Southern blot strategy
Restriction enzyme
Probe
Targeted
BamHI
5' Probe
9.88kb
KpnI
3' Probe
9.06kb
WT
5' probe
3' probe
9.88kb
9.06kb
C
NT
LPS
LPS + Nig
LPS+LPSe
Cre-
Cre+
kD
CASP1
Pro
p20
IL-1β
Pro
p17
Flag
Actin
55
40
25
15
35
D
GSDMD-N LSL
GSDMD-N LSL;LysM-CRE
Cytotoxity
LDH Release(%)
ns
*
***
NT
LPS
LPS+Nig
LPS+LPSe
E
GSDMD-N LSL
GSDMD-N LSL;LysM-CRE
**
Percent survival
Time after challenge with LPS(h)
0
12
24
36
48
60
100
80
60
40
20

Fig. S6. Engineering strategy to generate *GSDMD-N*^{LSL}; *LysM*-Cre mice, inflammasome activation of primary BMDMs from *GSDMD-N*^{LSL}; *LysM*-Cre mice and LPS-induced sepsis in these transgenic mice

(A) Targeting vector design for ROSA26 gene containing 3×Flag-GSDMD-N fragment with loxp-flanked stop cassette and the representative image for genotyping analysis of loxp sites.
(B) Regions selected for Southern blotting and the result of Southern blotting analysis.
(C) Immunoblot analysis of total cell lysates or the culture supernatants from primary BMDMs of *GSDMD-N*^{LSL}; *LysM*-Cre mice (cre+) and littermate control (cre-) after LPS-Nig (1h) stimulation and LPS electroporation (6h).
(D) LDH released from primary BMDMs of *GSDMD-N*^{LSL}; *LysM*-Cre mice and littermate control upon inflammasome activation as indicated.
(E) Survival of *GSDMD-N*^{LSL} and *GSDMD-N*^{LSL}; *LysM*-Cre mice in LPS (25mg/kg) -induced sepsis model (n=6).
Data are representative of three independent experiments (C) or are pooled from three independent experiments (D). *$p < 0.05$, **$p < 0.01$, ***$p <0.001$, ns, not significant. Two-way ANOVA with Sidak's multiple comparisons test for (D). Log rank (Mantel-Cox) test for (E).

A

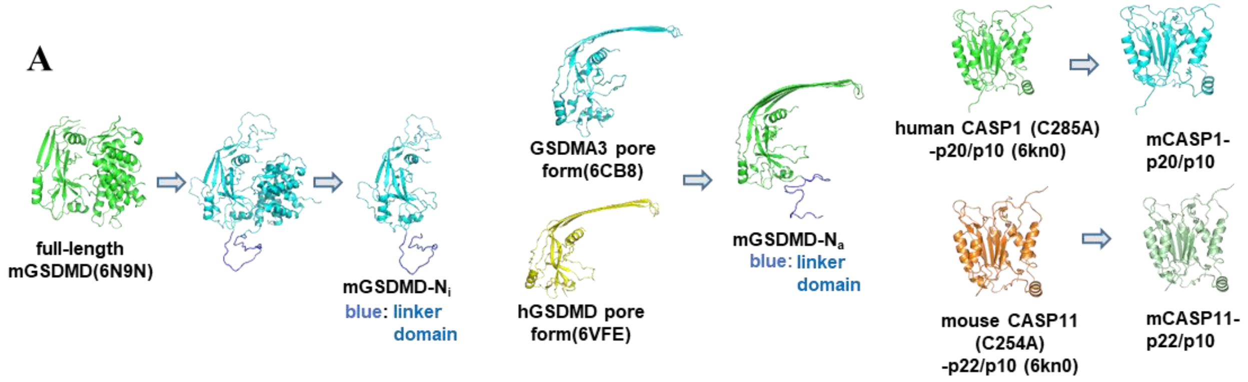

B

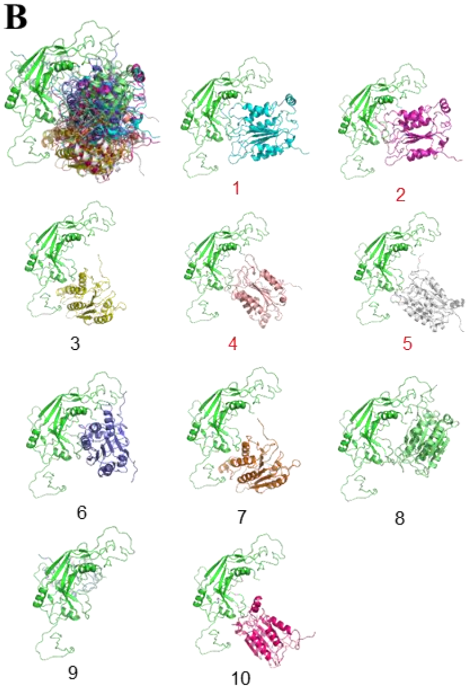

Top10	ZDOCK Score
1	1481.009
2	1330.361
3	1300.194
4	1276.654
5	1269.271
6	1253.032
7	1252.335
8	1251.38
9	1224.414
10	1216.645

C

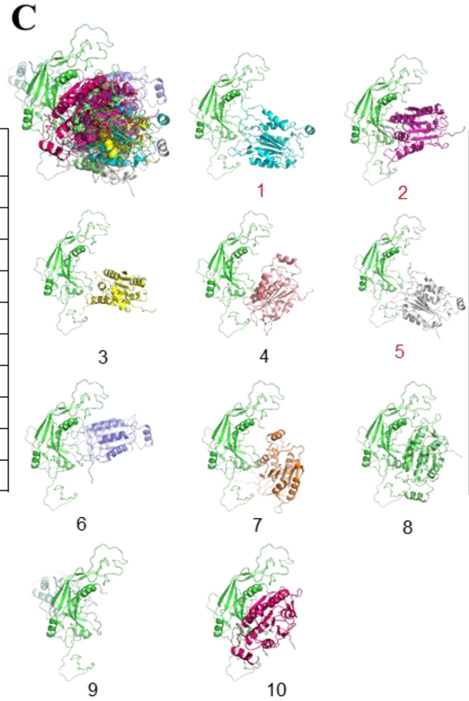

Top10	ZDOCK Score
1	1502.537
2	1377.77
3	1320.34
4	1263.61
5	1218.352
6	1211.571
7	1188.101
8	1180.157
9	1158.452
10	1108.849

D

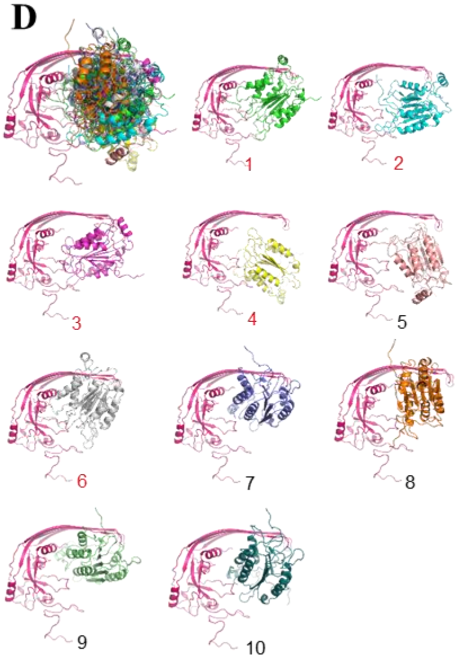

Top10	ZDOCK Score
1	1465.797
2	1433.354
3	1384.96
4	1309.784
5	1296.73
6	1289.993
7	1286.283
8	1285
9	1256.181
10	1255.169

E

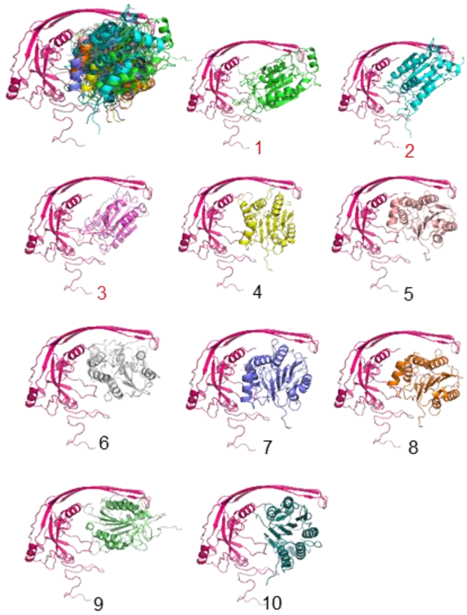

Top10	ZDOCK Score
1	1313.207
2	1281.773
3	1172.359
4	1157.609
5	1157.453
6	1154.98
7	1137.332
8	1131.34
9	1120.559
10	1119.605

Fig. S7. Homology-modeling and TOP 10 random docking structures

(A) The inactivating conformation of murine GSDMD-N fragment with cleavage linker (mGSDMD-N_i) based on the structure of full-length murine GSDMD (PDB: 6N9N) were generated by program Modeller, and the activating pore conformation of murine GSDMD-N fragment with flexible cleavage linker (mGSDMD-N_a) based on the pore structure of mGSDMA3 (PDB: 6CB8) and hGSDMD (6VFE). The structural model of murine CASP1 (p20/p10) was generated based on the structure of human CASP1 (C285A)-p20/p10 (PDB: 6KN0) by homology-modeling (SWISS-MODEL). The CASP11 (C254A) -p22/p10 structure (PDB: 6KMU) was used as murine CASP11 (p22/p10) model.

(B and C) Top 10 random docking structures and ZDOCK score of mGSDMD-N_i and CASP1 (p20/p10) (B) or CASP11 (p22/p10) (C).

(D and E) Top 10 random docking structures and ZDOCK score of mGSDMD-N_a and CASP1 (p20/p10) (D) or CASP11 (p22/p10) (E).

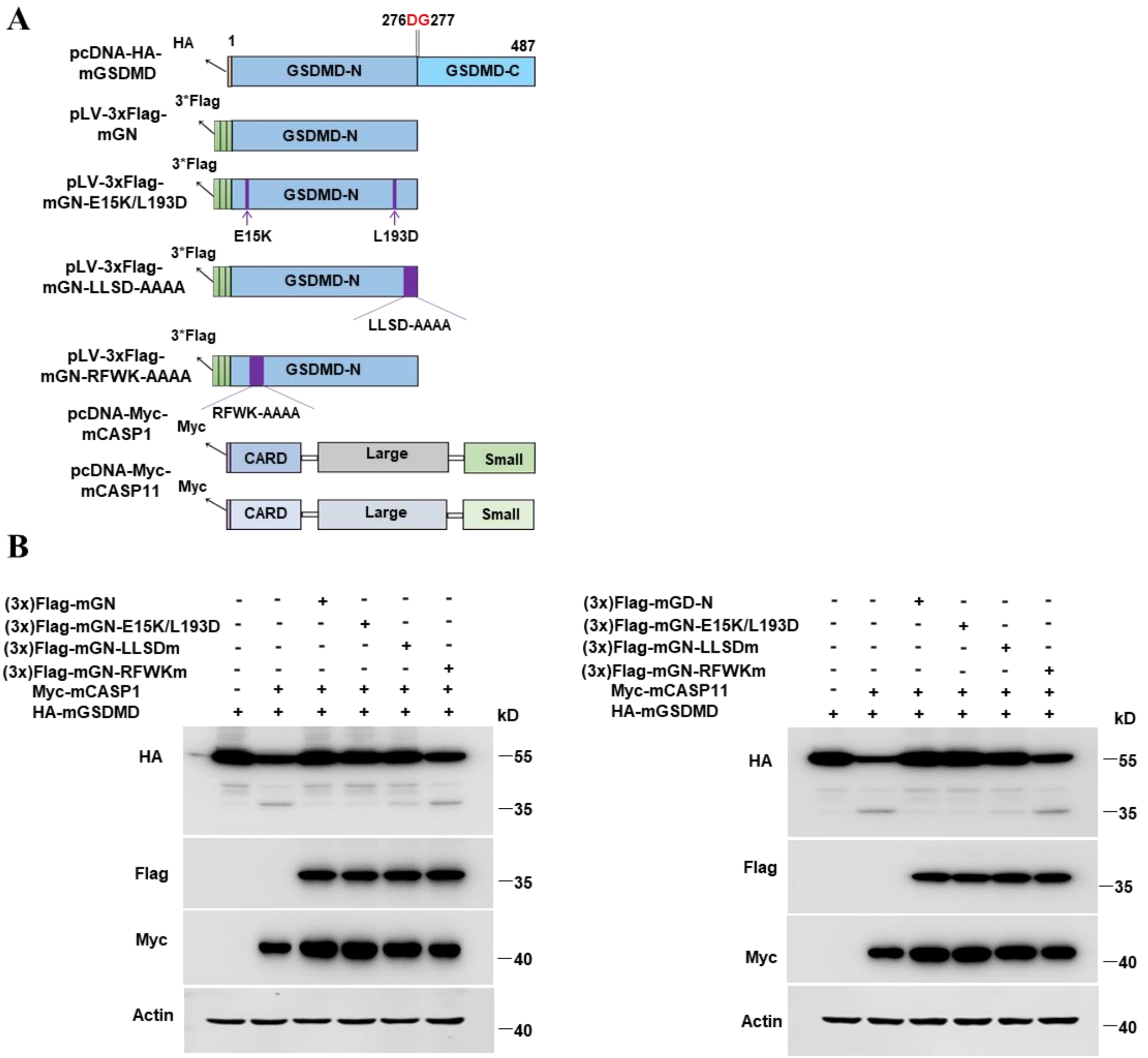

Fig. S8. The N-terminal fragment of GSDMD inhibits CASP1/11 activity via its RFWK motif in co-transfection system

(A) Domain architecture of the N terminal fragment and N terminal fragment mutants (mGSDMD-N_{RFWKm}, mGSDMD-N_{LLSDm} and mGSDMD-$N_{E15K/L193Dm}$) with 3×Flag tag at N terminus, Myc-tagged mouse CASP1/11(mCASP1/11) and HA-tagged full-length mouse GSDMD (HA-mGSDMD).
(B) Immunoblot analysis of HA-tagged mGSDMD cleaved by CASP1/11 in the presence of Flag-tagged mGSDMD-N and mGSDMD-N mutants (mGSDMD-$N_{E15K/L193Dm}$, mGSDMD-N_{LLSDm} and mGSDMD-N_{RFWKm}) by co-transfection of these constructs in HEK293Tcells.
Data are representative of three independent experiments.

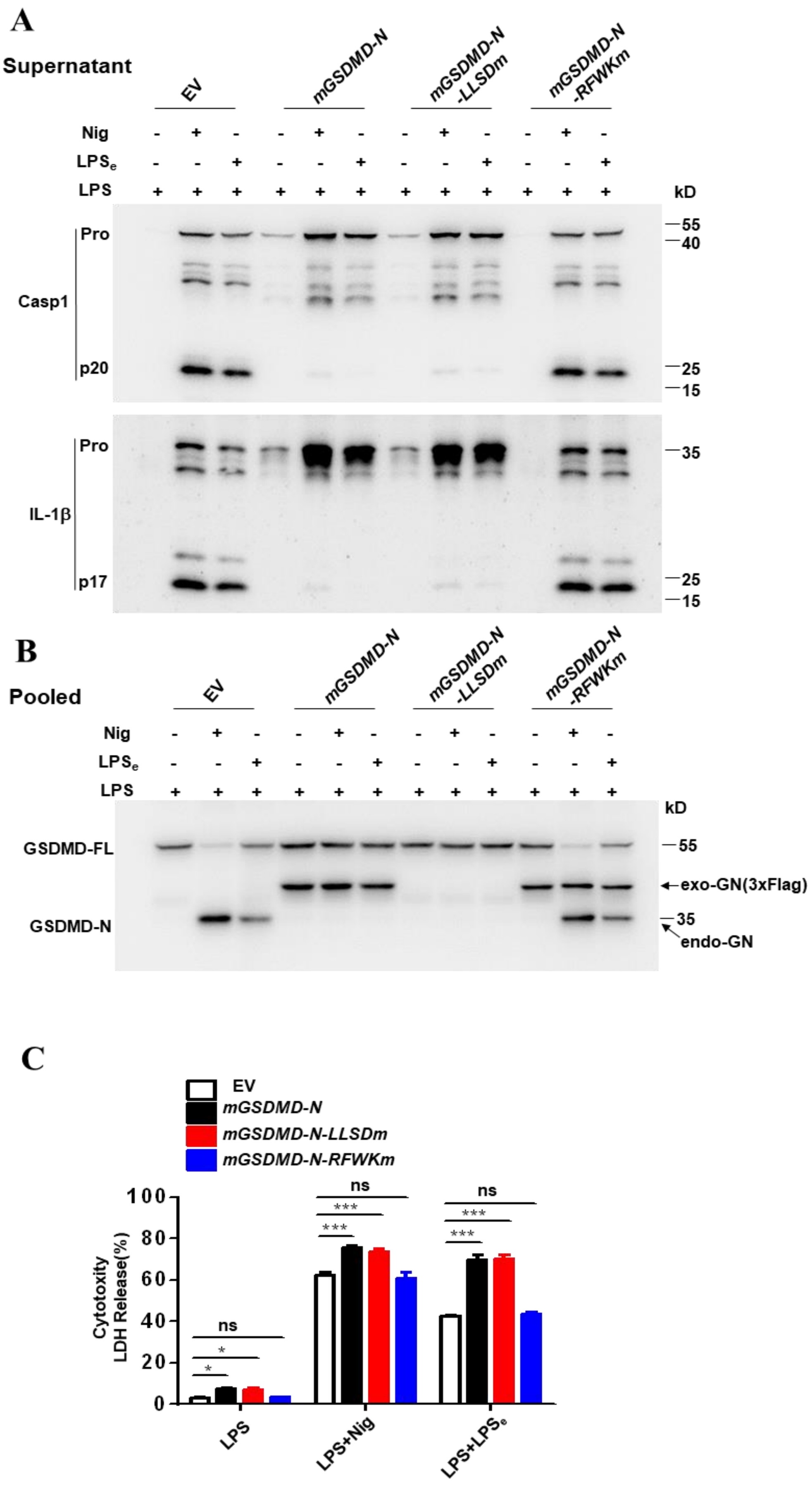
A
Supernatant
EV
mGSDMD-N
mGSDMD-N -LLSDm
mGSDMD-N -RFWKm
Nig
LPSe
LPS
kD
Pro
Casp1
p20
55
40
25
15
Pro
IL-1β
p17
35
25
15
B
Pooled
EV
mGSDMD-N
mGSDMD-N -LLSDm
mGSDMD-N -RFWKm
Nig
LPSe
LPS
kD
GSDMD-FL
GSDMD-N
55
exo-GN(3xFlag)
35
endo-GN
C
EV
mGSDMD-N
mGSDMD-N-LLSDm
mGSDMD-N-RFWKm
Cytotoxity
LDH Release(%)
100
80
60
40
20
0
ns
*
*
***
***
ns
***
***
ns
LPS
LPS+Nig
LPS+LPSe

Fig. S9. iBMDM cells stably expressing mGSDMD-N_{RFWKm} exhibit less inflammasome activation and LDH release in the supernatants after inflammasome activation

(A) Immunoblot analysis of supernatants from iBMDMs stably expressing mGSDMD-N, mGSDMD-N_{LLSDm} and mGSDMD-N_{RFWKm} during LPS-Nig stimulation and LPS electroporation.

(B) Immunoblot analysis of endogenous and exogenous GSDMD expression in pooled supernatants and cell extracts from iBMDMs stably expressing mGSDMD-N, mGSDMD-N_{LLSDm} and mGSDMD-N_{RFWKm} after LPS-Nig stimulation (1h) and LPS electroporation (6h). Endo-GN, endogenous GSDMD N-terminal fragments; exo-GN(3xFlag), exogenous GSDMD N-terminal fragments with 3xFlag tag.

(C) LDH released from iBMDMs stably expressing mGSDMD-N, mGSDMD-N_{RFWKm} and mGSDMD-N_{LLSDm} during LPS-Nig stimulation and LPS electroporation.

Data are representative of three independent experiments (A) or are representative of two independent expreiments (B) or are pooled from three independent experiments (C). *$p < 0.05$, ***p <0.001, ns, not significant. Two-way ANOVA with Sidak's multiple comparisons test for (C).

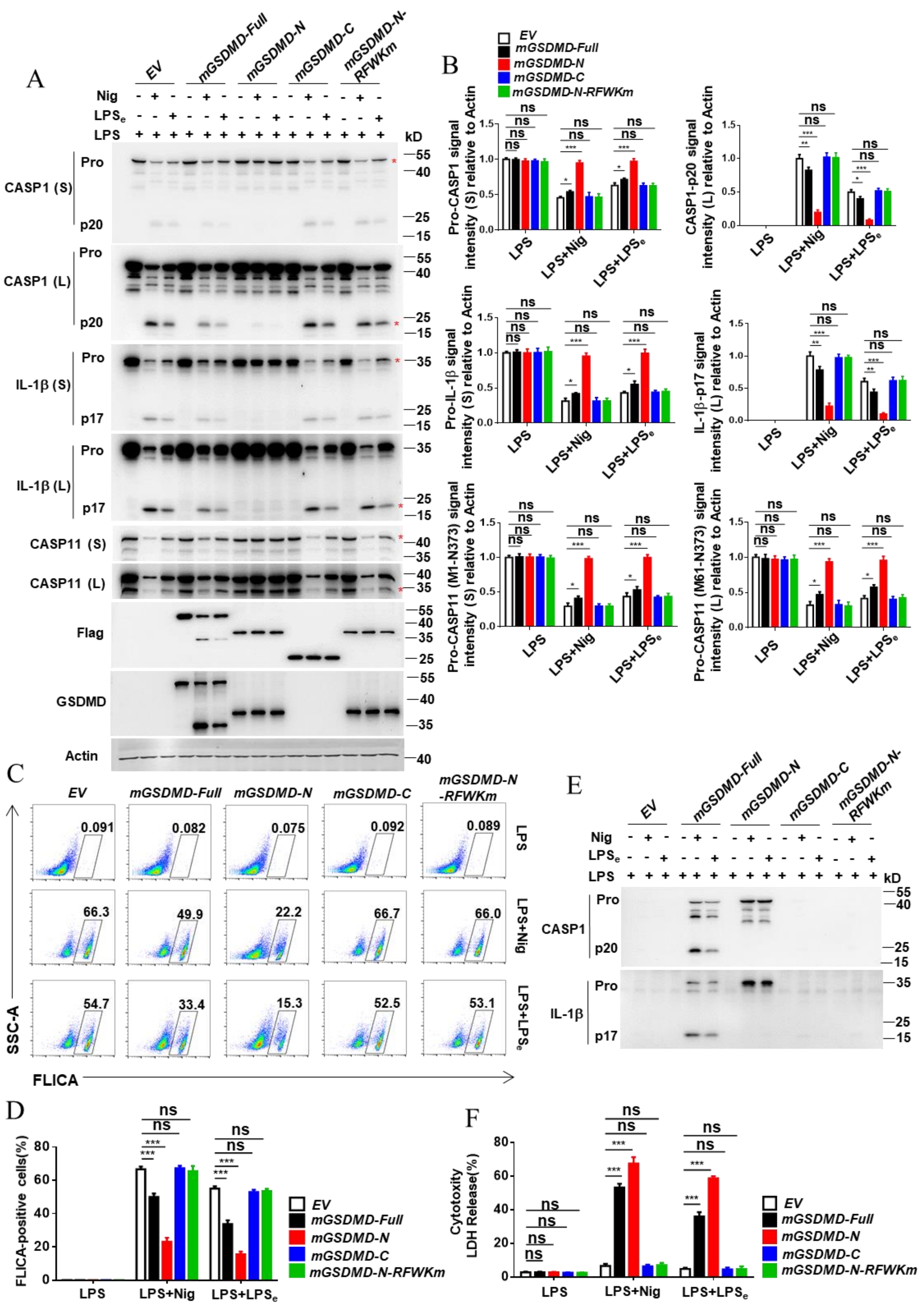
A
B
C
D
E
F
EV
mGSDMD-Full
mGSDMD-N
mGSDMD-C
mGSDMD-N-RFWKm
Nig
LPSe
LPS
kD
CASP1 (S)
CASP1 (L)
IL-1β (S)
IL-1β (L)
CASP11 (S)
CASP11 (L)
Flag
GSDMD
Actin
Pro
p20
p17
SSC-A
FLICA
LPS+Nig
LPS+LPSe
FLICA-positive cells(%)
Cytotoxicity LDH Release(%)
CASP1
IL-1β
ns

Fig. S10. Stable expression of mGSDMD-N in *GSDMD*$^{-/-}$ iBMDMs inhibits CASP1/11 activity during inflammasome activation

(A) Immunoblot analysis of pooled cell extracts and supernatants from *GSDMD*$^{-/-}$ iBMDMs stably expressing full length (mGSDMD-Full), N-terminal (mGSDMD-N), C-terminal (mGSDMD-C) and N-terminal RFWK mutant (mGSDMD-N-RFWKm) fragments of mouse GSDMD during canonical (LPS-Nig, Nig for 1 h) or non-canonical (LPS electroporation for 6 h) inflammasome-triggered processing of pro-CASP1/11 and pro-IL-1β.
(B) Relative quantification analysis based on grayscale values of (A). Referring to Actin, the signal intensity of pro-CASP1, pro-IL-1β and pro-CASP11(M1-N373) were calculated by the gray value of shorter exposure; the CASP1, IL-1β and pro-CASP11(M61-N373) were calculated by the gray value of longer exposure. * indicates the bands used for grayscale analysis.
(C and D) Flow-cytometric analysis of CASP1/11 activation probed by FLICA-positive staining in *GSDMD*$^{-/-}$ iBMDMs stably expressing mGSDMD-Full, mGSDMD-N, mGSDMD-C and mGSDMD-N-RFWKm upon inflammasome activation as indicated.
(E) Immunoblot analysis of supernatants from *GSDMD*$^{-/-}$ iBMDMs stably expressing mGSDMD-Full, mGSDMD-N, mGSDMD-C and mGSDMD-N-RFWKm upon inflammasome activation as indicated.
(F) LDH released from *GSDMD*$^{-/-}$ iBMDMs stably expressing mGSDMD-Full, mGSDMD-N, mGSDMD-C and mGSDMD-N-RFWKm upon inflammasome activation as indicated.
Data are representative of three independent experiments (A, C and E) and pooled from three independent experiments (B, D and F). Error bars show means ± SEM. $*p < 0.05$, $** p < 0.01$, $***p < 0.001$; ns, not significant. Two-way ANOVA with Sidak's multiple comparisons test for (B, D and F).

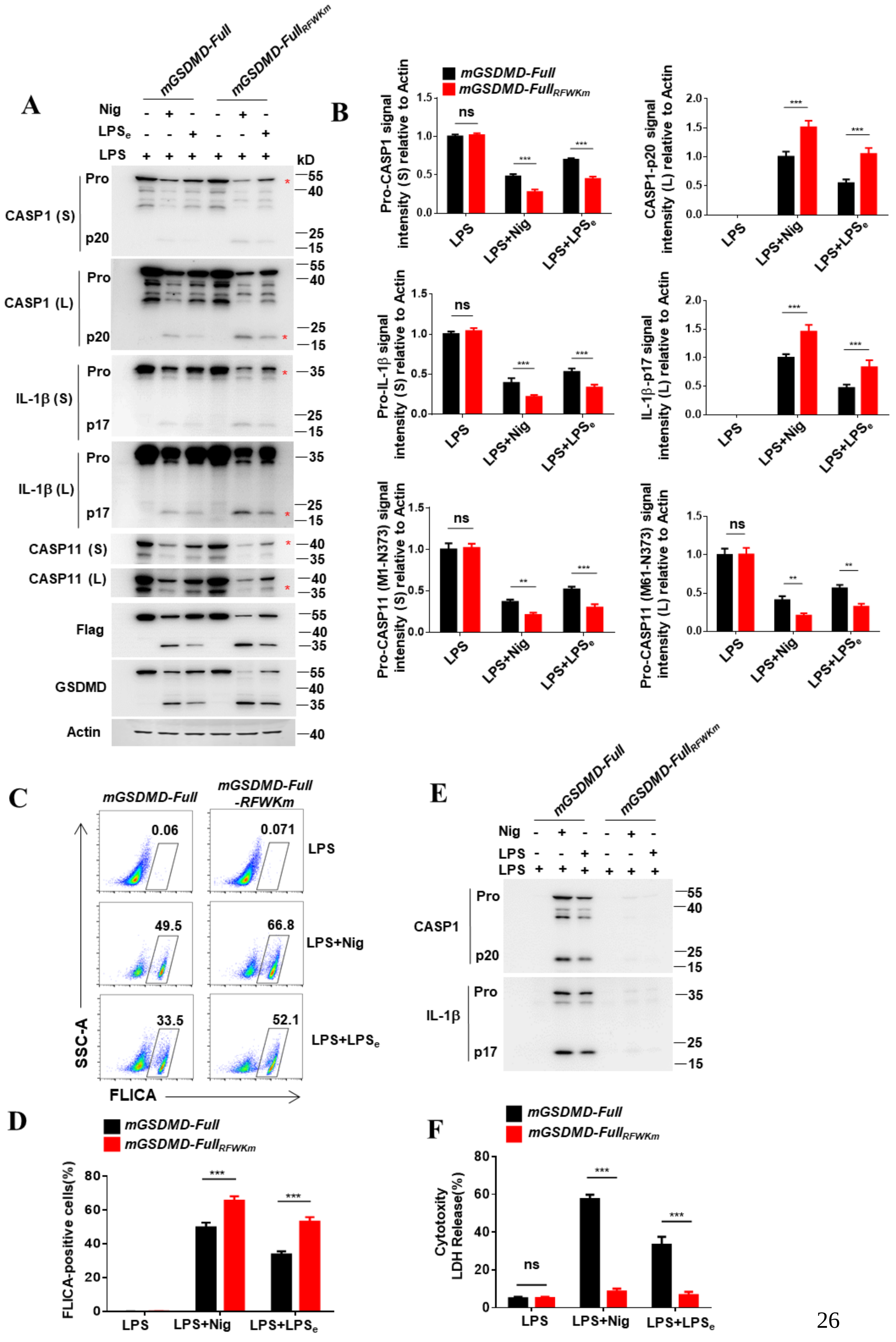
A
mGSDMD-Full
mGSDMD-Full RFWKm
Nig
LPSe
LPS
kD
CASP1 (S)
CASP1 (L)
IL-1β (S)
IL-1β (L)
CASP11 (S)
CASP11 (L)
Flag
GSDMD
Actin
Pro
p20
p17
B
mGSDMD-Full
mGSDMD-Full RFWKm
Pro-CASP1 signal intensity (S) relative to Actin
CASP1-p20 signal intensity (L) relative to Actin
Pro-IL-1β signal intensity (S) relative to Actin
IL-1β-p17 signal intensity (L) relative to Actin
Pro-CASP11 (M1-N373) signal intensity (S) relative to Actin
Pro-CASP11 (M61-N373) signal intensity (L) relative to Actin
LPS
LPS+Nig
LPS+LPSe
ns
C
mGSDMD-Full
mGSDMD-Full -RFWKm
0.06
0.071
49.5
66.8
33.5
52.1
LPS
LPS+Nig
LPS+LPSe
SSC-A
FLICA
D
FLICA-positive cells(%)
E
CASP1
IL-1β
F
Cytotoxicity LDH Release(%)

Fig. S11. Reconstituting GSDMD deficient iBMDMs with GSDMD full length RFWK mutant exacerbates inflammasome activation compared with GSDMD WT full length

(A) Immunoblot analysis of pooled cell extracts and supernatants from mGSDMD-Full$_{RFWKm}$ during canonical (LPS-Nig, Nig for 1 h) or non-canonical (LPS electroporation for 6 h) inflammasome-triggered processing of pro-CASP1/11 and pro-IL-1β.
(B) Relative quantification analysis based on grayscale values of (A). Referring to Actin, the signal intensity of pro-CASP1, pro-IL-1β and pro-CASP11(M1-N373) were calculated by the gray value of shorter exposure; the CASP1, IL-1β and pro-CASP11(M61-N373) were calculated by the gray value of longer exposure. * indicates the bands used for grayscale analysis.
(C and D) Flow-cytometric analysis of CASP1/11 activation probed by FLICA-positive staining in mGSDMD-FullRFWKm upon inflammasome activation as indicated.
(E) Immunoblot analysis of supernatants from mGSDMD-Full$_{RFWKm}$ iBMDMs in response to inflammasome activation.
(F) LDH released from mGSDMD-FullRFWKm upon inflammasome activation as indicated.
Data are representative of three independent experiments (A, C and E) or are pooled from three independent experiments (B, D and F). Error bars show means ± SEM. $^{**}p < 0.01$, $^{***}p < 0.001$; ns, not significant. Two-way ANOVA with Sidak's multiple comparisons test for (B, D and F).

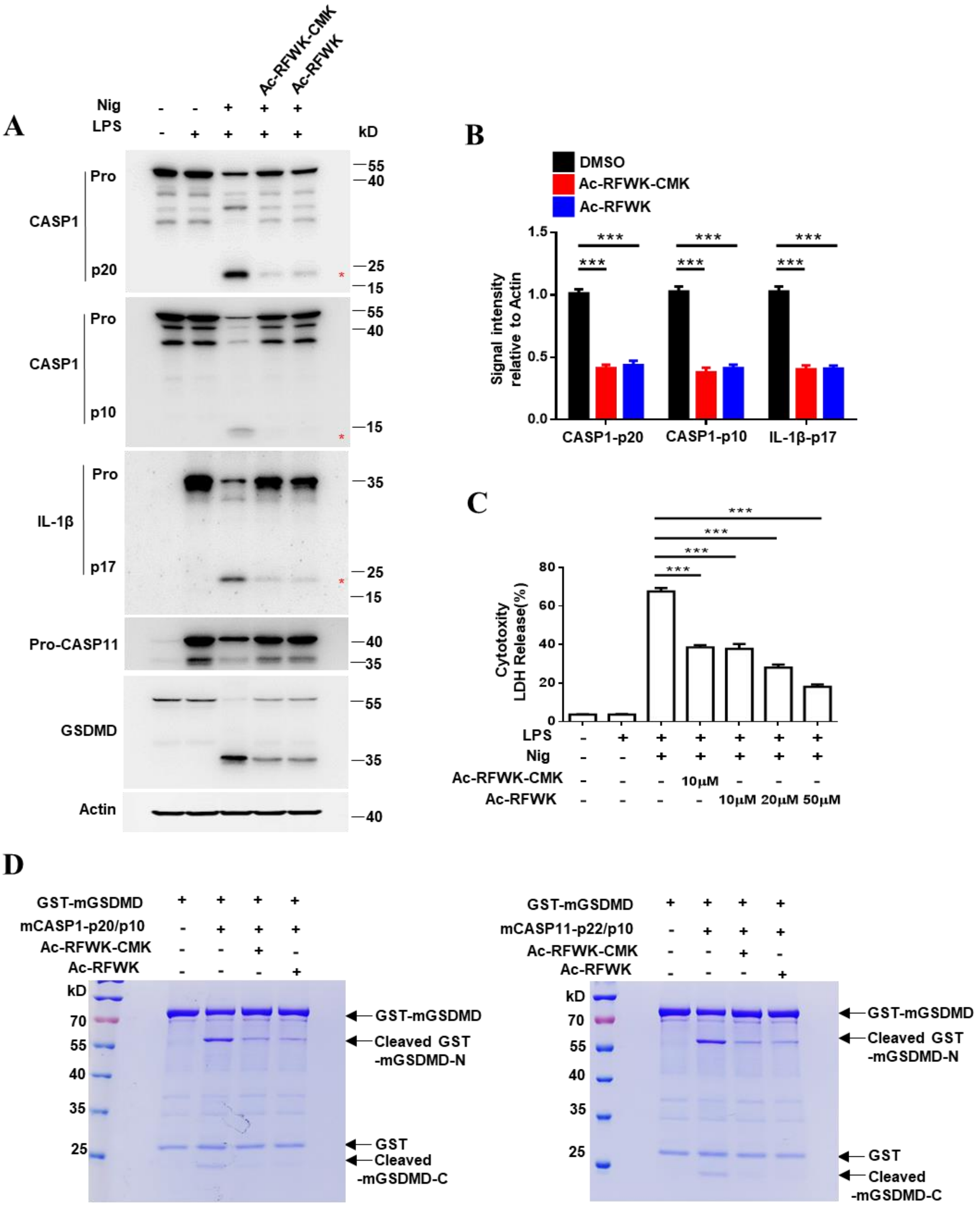
A
Nig
LPS
Ac-RFWK-CMK
Ac-RFWK
kD
CASP1
Pro
p20
CASP1
Pro
p10
IL-1β
Pro
p17
Pro-CASP11
GSDMD
Actin
B
DMSO
Ac-RFWK-CMK
Ac-RFWK
Signal intensity relative to Actin
CASP1-p20
CASP1-p10
IL-1β-p17
C
Cytotoxicity LDH Release(%)
LPS
Nig
Ac-RFWK-CMK
Ac-RFWK
10μM
10μM 20μM 50μM
D
GST-mGSDMD
mCASP1-p20/p10
Ac-RFWK-CMK
Ac-RFWK
GST-mGSDMD
Cleaved GST -mGSDMD-N
GST
Cleaved -mGSDMD-C
GST-mGSDMD
mCASP11-p22/p10
Ac-RFWK-CMK
Ac-RFWK
GST-mGSDMD
Cleaved GST -mGSDMD-N
GST
Cleaved -mGSDMD-C

Fig. S12. RFWK motif-based parent inhibitor Ac-RFWK suppresses inflammasome activation

(A) Immunoblot analysis of pooled cell extracts and supernatants from primary wild-type BMDMs in the presence of Ac-RFWK-CMK (10μM) and Ac-RFWK (10μM) after NLRP3 inflammasome activation (LPS-Nig, Nig for 1 h).

(B) Relative quantification analysis based on grayscale values of (A). * indicates the bands used for grayscale analysis.

(C) LDH released from wild-type BMDMs in the presence of Ac-RFWK-CMK and Ac-RFWK after NLRP3 inflammasome activation.

(D) Recombinant full-length GSDMD protein cleaved by CASP1/11 in the presence of Ac-RFWK-CMK (10μM) and Ac-RFWK (10μM) in vitro.

Data are representative of three independent experiments (A and D) or are pooled from three independent experiments (B and C). Error bars show means ± SEM. ***$p < 0.001$. Two-way ANOVA with Sidak's multiple comparisons test for (B and C).

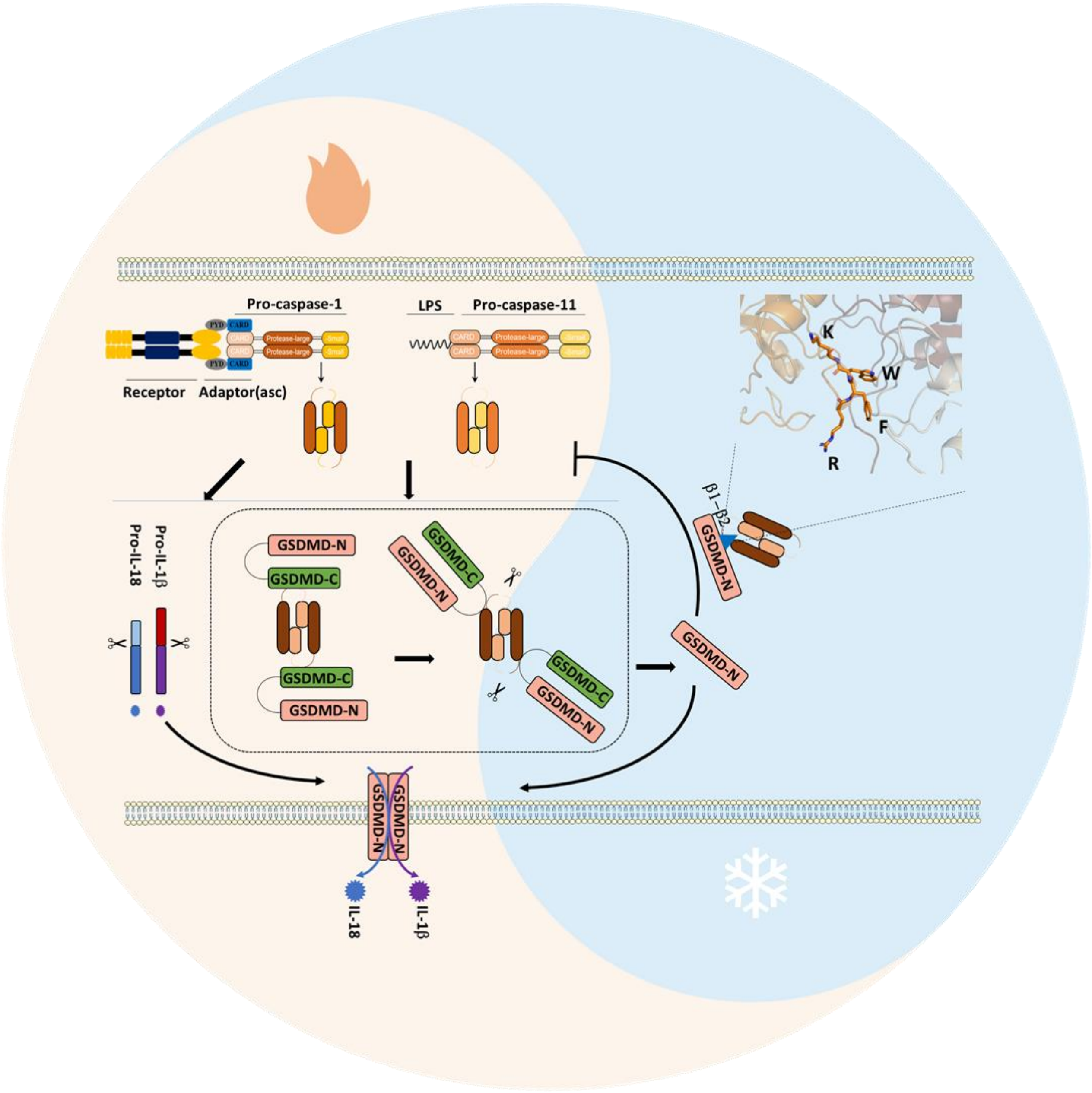

Fig. S13. Model for feedback inhibition of the activation of CASP1/11 through RFWK motif of the N-terminal fragment of Gasdermin D

Gasdermin D utilizes a negative feedback mechanism to inhibit Caspase-1/11 activation through its RFWK motif in N-terminal β1–β2 loop

References

1. C. L. Evavold *et al.*, Control of gasdermin D oligomerization and pyroptosis by the Ragulator-Rag-mTORC1 pathway. *Cell* **184**, 4495-4511 e4419 (2021).
